# Anesthetic protocol for microinjection-related handling of Siberian sturgeon (*Acipenser baerii*; Acipenseriformes) prolarvae

**DOI:** 10.1371/journal.pone.0209928

**Published:** 2018-12-31

**Authors:** Eun Jeong Kim, Yoon Kwon Nam

**Affiliations:** Department of Marine Bio-Materials and Aquaculture, Pukyong National University, Busan, South Korea; Harvard University Faculty of Arts and Sciences, UNITED STATES

## Abstract

An anesthetic protocol was optimized for microinjection-related handling of Siberian sturgeon (*Acipenser baerii*; Acipenseriformes) prolarvae, an extant primitive fish species commonly grown in aquaculture. Comparative examinations of three selected anesthetics (clove oil, lidocaine, and MS-222) with a dosage regime of 50, 100, 200, and 400 mg/L indicated that MS-222 was the most efficient agent for Siberian sturgeon prolarvae, as evidenced by the fast induction of anesthesia with quick and uniform recovery. Meanwhile, clove oil should be avoided, due to prolonged recovery times varying widely between individuals. None of the tested anesthetics significantly affected prolarval viability at any of the dosage regimes tested in this study. Based on an analysis of the duration of an unconscious state in air, we recommend a dose of 200 mg/L MS-222 for microinjection. Recovery time after use of this dose was influenced by the prolarval age and the development of gills, in which prolarvae older than 3 days after hatching required longer recovery times than did younger prolarvae. Post-recovery behavioral assessment showed no apparent difference between MS-222-anesthetized and non-anesthetized prolarvae in their swimming behavior and phototactic responses. Applicability of currently developed anesthetic protocol using MS-222 in larval microinjection was demonstrated with the injection of a visible dye to the anesthetized prolarvae, followed by the analysis of post-recovery viability. Taken together, the present anesthetic protocol based on 200 mg/L of MS-222 could provide researchers with practical usefulness with good safety margins for the micromanipulation and other related handlings of Siberian sturgeon prolarvae.

## Introduction

Anesthesia is generally defined as a reversible state resulting in unconsciousness and loss of sensation against external and internal stimulation, through the depression of the central nervous system. This state may be followed by different levels of analgesia and muscle relaxation [1, 2]. The use of adequate anesthetic protocols in fish research is advocated from an ethical perspective in promoting animal welfare and scientific perspectives [3]. However, efficacy of anesthetic agents should potentially be influenced by a number of biotic factors, including species, size, age, sex, and maturity, as well as abiotic factors such as temperature, water parameter, and salinity [1, 4]. Since no anesthetic agent is effective in all scenarios, the choice of anesthetic and dosage regimes should be determined based on experiment with each target species [2].

Microinjection is a technique to inject defined volumes and concentrations of substances, for purposes of exploring their physiological function *in vivo* [5]. Microinjection-based delivery of cells, nucleic acids, and/or other agents to fish larvae has been widely used in various investigations including molecular imaging, xenotransplantation, generation of germ-line chimera transgenics, infection, and gene knockdown assay [6, 7, 8]. During microinjection procedures, non-invasive handling and stable immobilization of small-sized fish larvae are needed, and thus, appropriate anesthetic treatment of the fish larvae is essential to not only ensure the stable operation of the injection but also to minimize pain and stress in the fish [9, 10].

Sturgeons belong to the Acipenseriformes (Chondrostei) order and subclass, an extant primitive fish group. Their evolutionary position relative to advanced bony fishes and other vertebrates makes them useful model system for evolutionary genomics with regard to the development and physiology of vertebrates [11, 12]. Unique or special features in their anatomy and physiology have also been attractive targets for researchers to identify novel genetic pathways and key genes that are potentially of biotechnological interest [13]. Recently, germ cell manipulation-based reproductive control of sturgeons has been one of key research issues with not only conservation aims, such as gene banking and restocking, but also aquaculture objectives such as shortening the maturation period through the germ-line chimera-mediated surrogate production. Microinjection is an essential tool in these investigations [7, 14, 15]. Applications of larval microinjection can also be expanded to various research fields of sturgeon biology so as to gain deeper insight into their molecular and physiological functions [16].

However, despite its importance, anesthetic protocols for sturgeon larvae have not been comprehensively established, and most postmortem studies on anesthesia have focused on juveniles, subadults, and adults. Based on this need, our study aimed to develop an anesthetic protocol for prolarvae (the yolk-bearing early larvae) of the Siberian sturgeon *Acipenser baerii*, one of the most widely cultured sturgeon species around the world, including in South Korea [17, 18]. For this, we evaluated efficacies of three anesthetics (clove oil, lidocaine and MS-222) with regard to suitable dosage regimes, anesthetic and recovery times, and effects on post-recovery viability and behavior. Microinjection of a visible dye into sturgeon prolarvae was also used to demonstrate the efficacy of the optimized anesthetic protocol for safe and non-invasive larval handling during microinjection procedures.

## Materials and methods

### Ethics statement

Described experiment was approved by the Animal Care and Use Committee of Pukyong National University (Approval number 201817). All experimental procedures were performed in accordance with National Act on Laboratory Animals.

### Fish specimens and rearing conditions

Gametes from Siberian sturgeon *Acipenser baerii* broodfish (two females and two males) were obtained using injected luteinizing hormone-releasing hormone analogue (LHRH-a; Sigma-Aldrich, Saint Louis, MO, USA) as per Park et al. [17]. Fertilized eggs were incubated at 20 ± 0.5°C until they hatched. Prolarvae hatched within 4 h of one another were transferred to a prolarval rearing tank, with conditions maintained according to Park et al. [17]. Briefly, approximately 5,000 prolarvae were incubated in each rectangular, white, polypropylene (PP) 2 × 1.2 × 0.4 m tank equipped with an external filter unit of 200-L capacity filled with 350 L of 1 μm-filtered groundwater. Incubation temperature was adjusted to 20 ± 0.5°C throughout the experiment, with pH at 7.2–7.6, dissolved oxygen at 7.5 ± 0.5 mg/L, and total ammonia nitrogen at 0.01–0.02 mg/L. Daily changes in body weight and total length during the prolarval period were measured to the nearest 0.1 mg and 0.1 mm. Analyses of morphological and behavioral ontogeny were performed based on descriptions by Gisbert and Nam [19].

### Preparation of anesthetic solutions

All chemicals used in this study were purchased from Sigma-Aldrich. Anesthetic agents included clove oil (1.04 g/mL at 25°C), lidocaine hydrochloride, and ethyl 3-aminobenzoate (MS-222). Stock solution for each anesthetic agent was made at 5 g/L using sterile, 18-megohm-cm deionized water. Clove oil was first dissolved in six volumes of 95% ethanol solution prepared with absolute ethyl alcohol in order to facilitate mixing with water, then diluted with deionized water to prepare stock solution. MS-222 was buffered to pH 7.2–7.4 using sodium bicarbonate. Anesthetic working solutions were prepared by diluting stock solutions with the 1 μm-filtered groundwater used in the larval rearing (see above) to adjust the final anesthetic dose to nominal concentration. Physico-chemical parameters of water used for anesthesia and recovery are provided in S1 Table. Working solutions were freshly prepared prior to all anesthesia treatments, with doses of 0, 50, 100, 200, or 400 mg/L tested for each agent.

### Anesthesia and recovery assessments

#### Common conditions for anesthesia and recovery

Throughout the anesthesia and recovery experiments, water temperature was adjusted to 19–20°C. Anesthetic solutions and water for recovery were exchanged for each replicate observation. For anesthetic induction, a plastic container (10 × 15 × 7 cm) containing 500 mL of anesthetic solution or filtered water for controls was used in each treatment. Stage of anesthesia was judged by total loss of equilibrium and swimming ability of prolarvae, while stage of recovery was determined by the beginning of locomotory swimming. After induction of anesthesia, prolarvae were transferred to a custom-designed recovery tank. The recovery tank (25 × 15 × 8 cm) contained 1.5 L water with dissolved oxygen levels averaging 7.3 ± 0.4 mg/L. During the recovery period, 500 lux light illumination was provided from above the tank. After transfer to the recovery tank, time to recovery was recorded for individual fish. Each recovered prolarva was immediately removed from the recovery tank and transferred to a net cage (20 × 15 × 15 cm) installed in a rearing tank as described above to monitor post-recovery survival.

#### Experiment A: Comparison of anesthetics and doses

Dose-dependent patterns of anesthetic and recovery times were compared among the three anesthetic agents with Day 0 prolarvae. Average body weight and total length of *A*. *baerii* prolarvae on Day 0 were 13.8 ± 0.6 mg and 9.8 ± 0.3 mm, respectively (S2 Table). Twenty fish were immersed in each anesthetic solution at each of the four prepared doses, plus control. Three replicates were performed for each treatment. Upon reaching anesthesia, prolarvae were transferred to the recovery tank, and elapsed times of 10% (R10), 50% (R50), and 100% (R100) of fish recovered were measured. After transferred to a rearing, the survival rate of prolarvae for each replicate group was examined at 1 h, 6 h, 12 h, and 24 h post-recovery.

#### Experiment B: Validation of efficacy of a selected anesthetic

In the Experiment B, the efficacy of MS-222 (selected based on the results from Experiment A) was further investigated by examining anesthetic and recovery times. In this experiment, anesthesia and recovery were examined in each individual, rather than in replicate groups. Each individual prolarva was immersed in 0, 50, 100, 200 or 400 mg/L of MS-222 to record time to anesthetic induction, then transferred to a recovery tank to record time to recovery. Thirty-six prolarvae were tested individually at each dosage. All other test conditions were as described in the Experiment A.

#### Experiment C: Test for the duration of anesthesia in room air

Two selected doses of MS-222 (100 and 200 mg/L) were further tested for the duration of anesthesia in air, since anesthetized prolarvae are usually exposed to the air during microinjection procedures. In order to reflect the actual conditions of microinjection, ten Day 0 or Day 1 prolarvae anesthetized with either 100 mg/L or 200 mg/L of MS-222 were placed on a 1% agarose bed and observed for 5 min, since this duration reflects the realistic timing for conventional microinjection of 7–10 larvae. Under a low magnification stereozoom microscope, fish were examined for reaction to external stimuli by gently pressing the boundary region between the prolarval body and yolk extension (i.e., the site for microinjection in this study) with a microinjection needle at 30 s intervals. All reflex activity against the external stimulus was recorded. After 5 min, all prolarvae were transferred to a recovery tank for monitoring recovery time. Viability was also examined at 1 h, 6 h, 12 h, and 24 h after recovery. Five replicate examinations were assigned to each concentration of MS-222.

#### Experiment D: Effects of prolarval ages on the anesthesia

We tested whether the age of prolarvae (Day 0-Day 5) influenced anesthetic/recovery times and post-recovery viability. Fish (N = 20 per replicate) from each age group were anesthetized with 200 mg/L of MS-222, and times to anesthesia and recovery times (R10, R50 and R100) were determined based on three replicate examinations. Information on length and weight of prolarvae is provided in S2 Table. All other test conditions were as described above.

#### Experiment E: Post-recovery behavioral assessment

Behavioral patterns of prolarvae recovered from anesthesia with MS-222 were compared with those of non-anesthetized control fish in order to examine whether the anesthetic treatment might cause changes in behavior. Because Siberian sturgeon prolarvae display light- and age-dependent behavioral modifications [20], we tested behavioral patterns both under daylight and dark conditions on Day 1, Day 2 and Day 4. On each day, 120 prolarvae were anesthetized with MS-222 at 200 mg/L, exposed to air for 5 min, and allowed to recover as described above. Recovered fish were transferred to one of three replicate examination tanks (0.6 × 0.5 × 0.25 m). Three replicate tanks for the non-anesthetized control groups were also prepared, each containing 120 non-anesthetized prolarvae (N = 120). Each tank contained approximately 60 L of 1-μm filtered groundwater and was equipped with a custom-designed 2-L filter unit. Water was re-circulated through the filter with a flow rate of 1 L/min. Under 500 lux in average adjusted with a white fluorescent room light, swimming behaviors were addressed based on the criteria described by Gisbert and Nam [19] and Gisbert et al. [20]. These include the drifting behavior (i.e., actively swimming up to the water surface and then passively sinking to the bottom), preference of water column (pelagic swimming in the upper water column or benthic movement across the tank bottom) and presence of rheotactic behavior. The same parameters (drifting, water column, and rheotaxis) were also examined under dark conditions (<1 lux) with the aid of an infrared lightening apparatus. In addition, phototactic response to a spotlight using a white light emitting diode bulb was also examined under dark conditions, since the prolarvae of this sturgeon species should display strong positive phototaxis during this stage of development [19, 21]. Spotlight illumination was placed at one corner of the tank 20 cm above the water surface in order to provide an average light intensity of 5,000 lux at the water surface below the spotlight. After the spotlight was switched on, the number of positively phototactic prolarvae gathering within 2 min at the corner in a 25 × 25 cm area was counted to evaluate the relative strength of phototaxis. Behavioral patterns of experimental groups and control groups were compared under daylight conditions at 0.5 h post recovery and under dark conditions at 1 h for general swimming behaviors and 1.5 h for phototactic response to a spotlight post-recovery. At each detection point, each replicate tank was examined at least eight times.

### Microinjection of larvae

*A*. *baerii* prolarvae were anesthetized with using 200 mg/L concentration of MS-222 and placed on a 1% agarose bed. Microinjection needles of 20 μm diameter were prepared from glass capillaries (World Precision Instruments, Sarasota, FL, USA) with a PC-10 Micropipette puller (Narishige, Tokyo, Japan) and EG-401 Micro grinder (Narishige). Microinjection was performed manually with a M-152 micromanipulator (Narishige). Injections were made at the border region between the prolarval body and the yolk extension [15]. The injection was traced using a visible methylene blue staining dye (Sigma-Aldrich) at 0.5 mg/mL. The amount of dye solution injected into each prolarva was estimated to be approximately 15–20 nL depending on injection batches. A group of 7–9 prolarvae was anesthetized for each trial, with eight replicate trials made to yield a dataset of 60 microinjected prolarvae. These trials were carried out at three different age stages (Day 0 to Day 1, Day 2, and Day 3). After injection, prolarvae were transferred to the recovery tank and then to rearing cages for monitoring post-procedure viability for up to 120 h post injection, at 12-h intervals. Two control groups, including an anesthetized but not-injected group, and a non-anesthetized group were also prepared on each day of treatment.

### Statistics

Differences in anesthetic and recovery times, duration of anesthesia, and post-recovery assessments were assessed using Student’s *t*-test and/or ANOVA followed by Tukey’s post hoc test at the level of P = 0.05. Coefficient of determination (R^2^) values were estimated in order to examine relationship between anesthetic doses and induction/recovery times.

## Results

### Anesthetic induction with different doses of clove oil, lidocaine and MS-222

**Fig 1:**
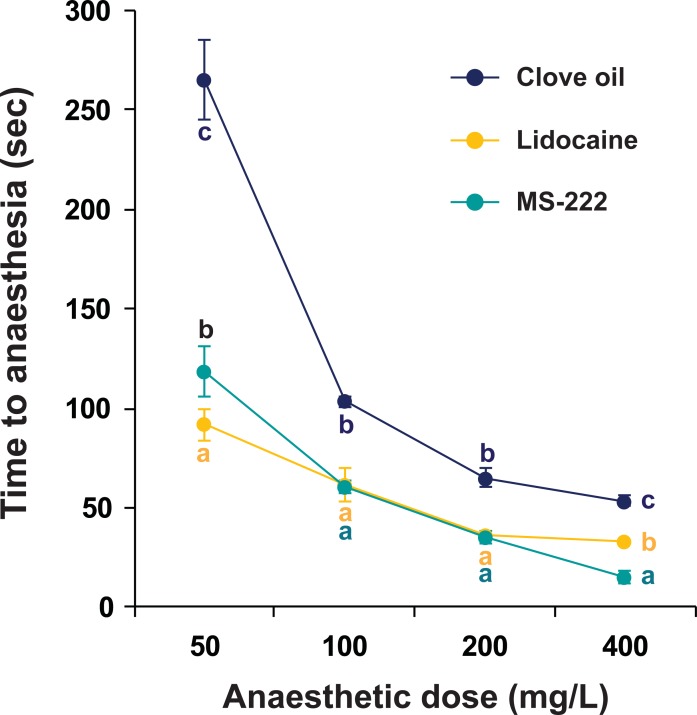
Anesthetic induction times of Siberian sturgeon prolarvae with clove oil, lidocaine, and MS-222. For each anesthetic, times to reach a stage of anesthesia (defined as total loss of equilibrium and swimming ability) were evaluated with four dose levels (50, 100, 200 and 400 mg/L). Triplicate examinations were made (N = 20 per replicate treatment). Mean ± SD with different letters (a-c) at each dose level indicate the statistical difference at P < 0.05 based ANOVA followed by Tukey’s post hoc test. Anesthetic induction time with 200 mg/L of MS-222 was further compared to those with increased concentrations (200–1600 mg/L) of lidocaine (S1 Fig).

In Experiment A, anesthetic times were inversely related to treatment concentrations (50 to 400 mg/L) of each anesthetic agent (Fig 1). Prolarvae treated with clove oil reached anesthesia after 265.0 ± 20.0 s, 103.3 ± 2.9 s, 65.0 ± 5.0 s, and 53.3 ± 2.9 s, at 50, 100, 200, and 400 mg/L dosages, respectively. Anesthetic times with lidocaine treatment were 91.7 ± 12.6 s, 6.7 ± 2.9 s, 36.7 ± 2.9 s, and 33.3 ± 2.9 s. MS-222 also showed dose-dependent decrease of anesthetic times, with 118.3 ± 7.6 s observed at 50 mg/L and 15.0 ± 0.0 s observed at 400 mg/L. Dose-dependency was most pronounced in MS-222 (R^2^ = 0.9354) and weakest in clove oil (R^2^ = 0.7882). Clove oil showed longer times to induction of anesthesia times than either lidocaine and MS-222 at every dose strength (P < 0.05). Between lidocaine and MS-222, time to induction at 50 mg/L was shorter in the lidocaine-treated groups than in those treated with MS-222 (P < 0.05). At 100 and 200 mg/L, the two anesthetics showed similar times to induction (P > 0.05). However, at the highest dose (400 mg/L), time to induction with MS-222 was significantly shorter than with lidocaine (P < 0.05).

### Patterns of recovery from anesthesia and larval viability

**Fig 2:**
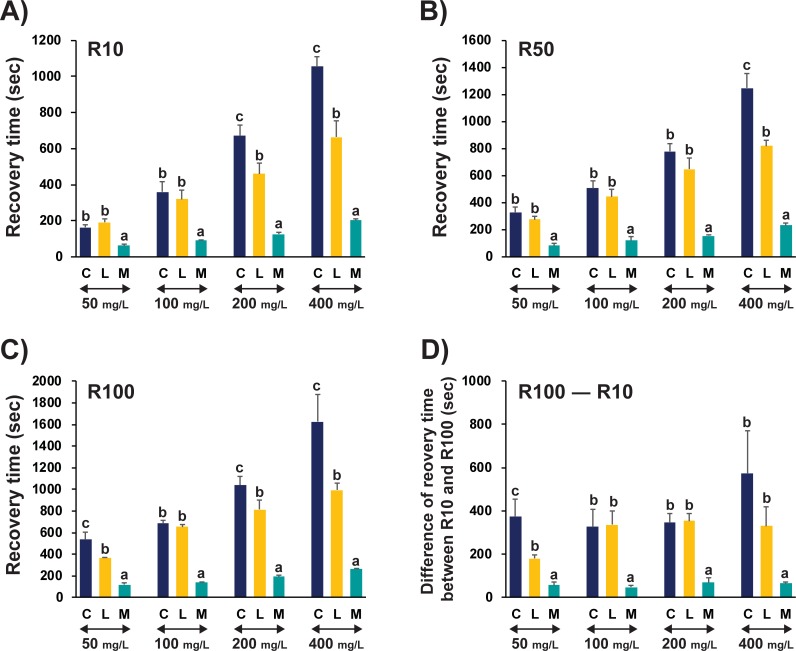
Recovery patterns of Siberian sturgeon prolarvae after anesthetic immersion treatment with clove oil (C), lidocaine (L) or MS-222 (M). Upon transfer to recovery tanks, times to recovery of 10% (**A**; R10), 50% (**B**; R50), and 100% (**C**; R100) of fish in each group were measured. Recovery was judged based on the beginning of locomotory swimming of the prolarvae. Difference of recovery times between R10 and R100 is indicated in (D). Within a given dose level, mean ± SD with different letters (a-c) indicate statistical difference among anesthetics at P < 0.05 based ANOVA followed by Tukey’s post hoc analysis. Triplicate examinations were made (N = 20 per replicate treatment). Statistical evaluations of R10, R50, and R100 values within a given anesthetic dose of each anesthetic agent are provided in S2 Fig. Data on post-recovery survival are provided in S3 Table. Recovery times after anesthetic induction with high concentrations of lidocaine (200–1600 mg/L) are also compared to that with 200 mg/L of MS-222 (S3 Fig).

Recovery times were positively related to anesthetic doses for each anesthetic agent irrespective of elapsed times to R10 (recovery of 10% prolarvae), R50 (50%) and R100 (100%) (Fig 2, S2 Fig). For R10, prolarvae anesthetized with MS-222 showed shorter recovery times than did other two anesthetics at all the dose strengths (P < 0.05) (Fig 2A). Clove oil and lidocaine showed similar R10 recovery times at 50 mg/L and 100 mg/L doses (P > 0.05). However, at higher doses (i.e., 200 mg/L and 400 mg/L), prolarvae anesthetized with clove oil required longer times for R10 recovery than did those anesthetized with lidocaine (P < 0.05). R50 recovery followed a similar pattern to R10, although a statistical difference in recovery time between clove oil and lidocaine was observed only at the highest dose (400 mg/L) (Fig 2B). For R100, differences in recovery time were more obvious among anesthetics. Statistical differences were detected at every dose strength (P < 0.05) except for at 100 mg/L, where there was no significant difference between clove oil and lidocaine (P > 0.05) (Fig 2C). As shown in Fig 2D, the time difference between R10 and R100 was significantly smaller in the group anesthetized with MS-222 than those treated with the other anesthetics. Although there were large differences in anesthetic and recovery times among anesthetics, prolarval viability was not significantly affected by the anesthetic agent used; survival rate post-anesthesia/recovery was 98% and more under all the treatment conditions (S3 Table).

### Individual variation in MS-222-mediated anesthesia

**Fig 3:**
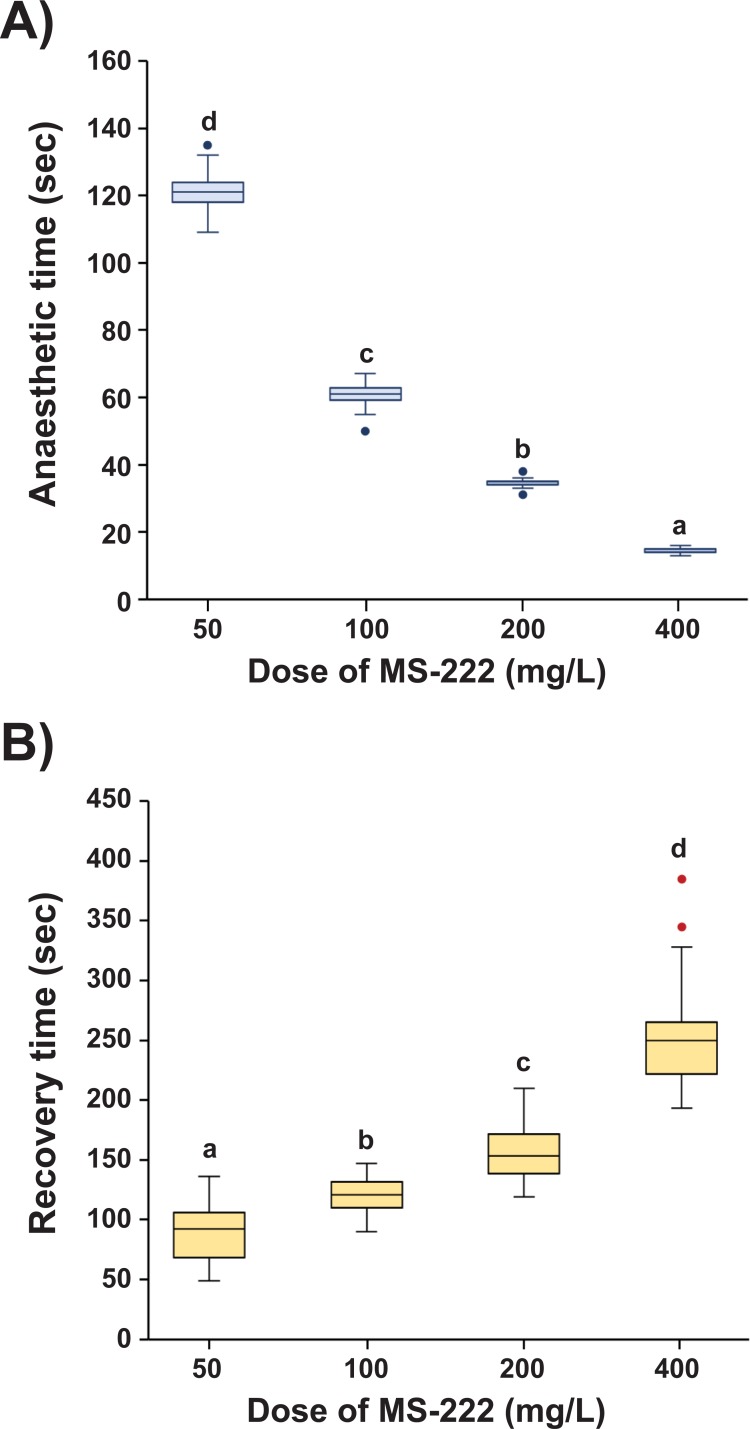
**Validation of anesthetic (A) and recovery (B) times with different doses of MS-222 on an individual-basis examination.** In the box plot, the lower and upper boundaries of each box indicate the 25th and 75th percentiles, respectively (N = 36). A line within the box marks the median value. Whiskers above and below the box indicate the 90th and 10th percentiles, respectively. Outliers are indicated by dots above and/or below the box. Mean ± SD with different letters (a-c) at each dose level indicate the statistical difference at P < 0.05 based ANOVA followed by Tukey’s post hoc analysis.

Examinations of anesthetic and recovery times with MS-222 in an individual manner (N = 36 prolarvae per dose group; Experiment B) reproduced the pattern observed in Experiment A (Fig 3). The induction time to anesthesia was inversely related with the dose of MS-222 (R^2^ = 0.9278), while longer recovery time was required under treatment with higher doses (R^2^ = 0.9231). Coefficients of variation for induction time in 50 mg/L-treated (CV = 0.043) and 100 mg/L-treated (CV = 0.064) groups were larger than for groups treated with higher doses (CV = 0.037 and 0.038, for 200 and 400 mg/L, respectively). The coefficient of variation for recovery time in the 200 mg/L-treated group (CV = 0.146) was lowest, followed by the coefficient of variation for the 100 mg/L (CV = 0.154), 400 mg/L (CV = 0.161) and 50 mg/L (CV = 0.264) treated groups. A few individuals treated with the highest dose showed exceptionally delayed recovery times.

### Anesthetic duration in air and response to external stimuli

**Fig 4:**
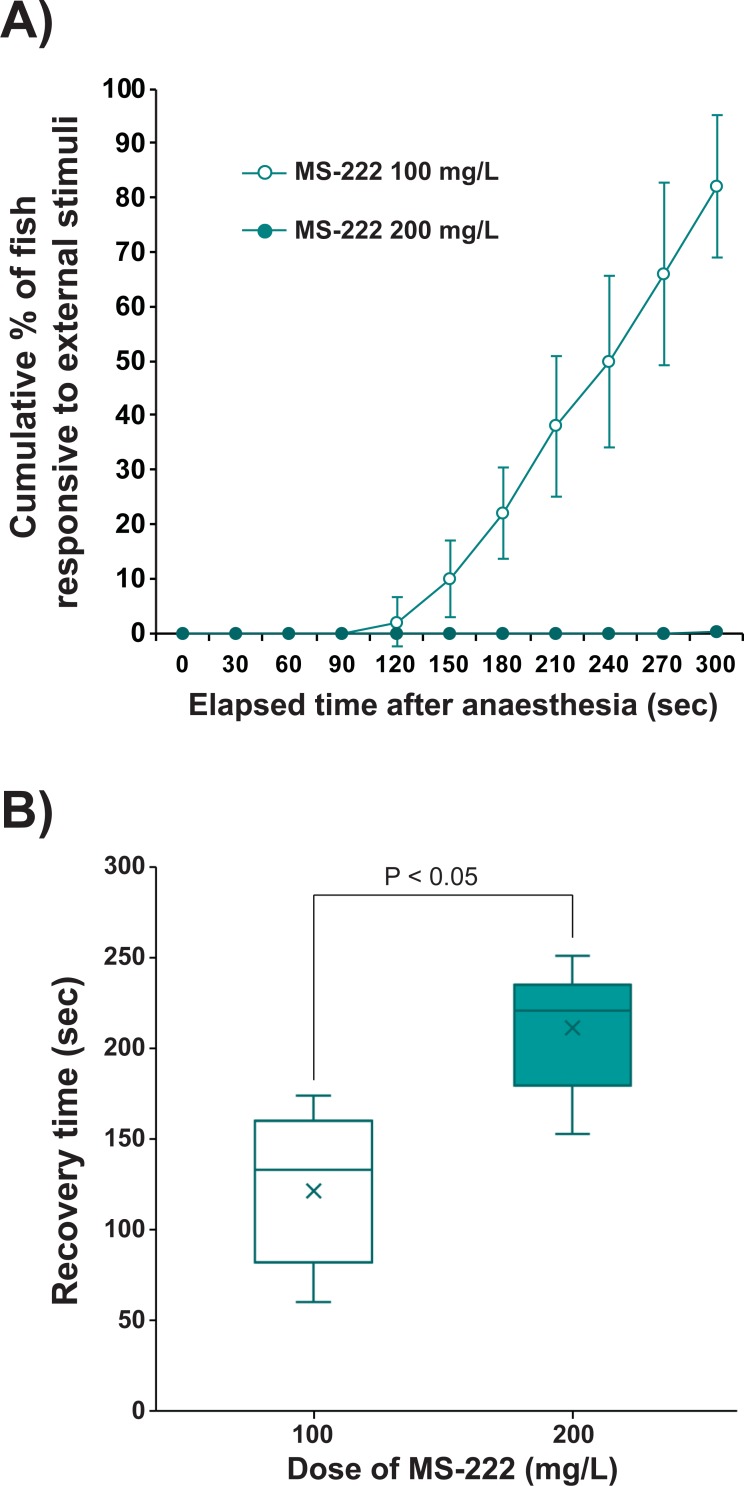
Reflex activity against external stimuli of prolarvae immersion treated with 100 and 200 mg/L of MS-222. Fish were anesthetized with 100 mg/L or 200 mg/L of MS-222, then exposed to air for 5 min. During air exposure, fish (N = 10 per replicate examination for either dose) were stimulated externally by gentle pressing with a glass needle. Five replicate examinations were made. (A) Cumulative percentage of fish showing response to external stimuli. (B) Recovery time after anesthetic treatment followed by 5-min air exposure. In B), lower and upper boundaries of each box indicate the 25th and 75th percentiles, respectively. A line within the box marks the median value. Mean value for each group is noted by the symbol ×. Whiskers above and below the box indicate the 90th and 10th percentiles, respectively. Similar with MS-222, lidocaine (200 mg/L) also displayed stable anesthesia in air without reflex activity against the same external stimuli, however, prolonged recovery time and mortality of a few individuals were observed (S4 Fig).

Anesthetic duration in the air without reflex activity against external stimuli differed between the doses of 100 mg/L and 200 mg/L of MS-222 (Experiment C). Neither group showed any response to external stimuli (i.e., gentle touch using a glass needle) until 90 s after the induction of anesthesia. However, from the point, the 100 mg/L-treated group showed a continuous increase in responsive individuals. Consequently, the cumulative percentage of responsive individuals in the 100 mg/L-treated group was 82.0 ± 13.0% at the end of observation at 300 s post-anesthesia. By contrast, prolarvae anesthetized with 200 mg/L were consistently non-responsive to the same external stimuli throughout the observation period (Fig 4A). As expected, recovery time after air exposure for 300 s was longer in the 200 mg/L-treated group (211.4 ± 32.7 s) than in the 100 mg/L-treated group (121.5 ± 38.7 s) (P < 0.05) (Fig 4B). No mortality was observed in group (S3 Table).

### Effect of fish age on anesthetic and recovery times with MS-222

**Fig 5:**
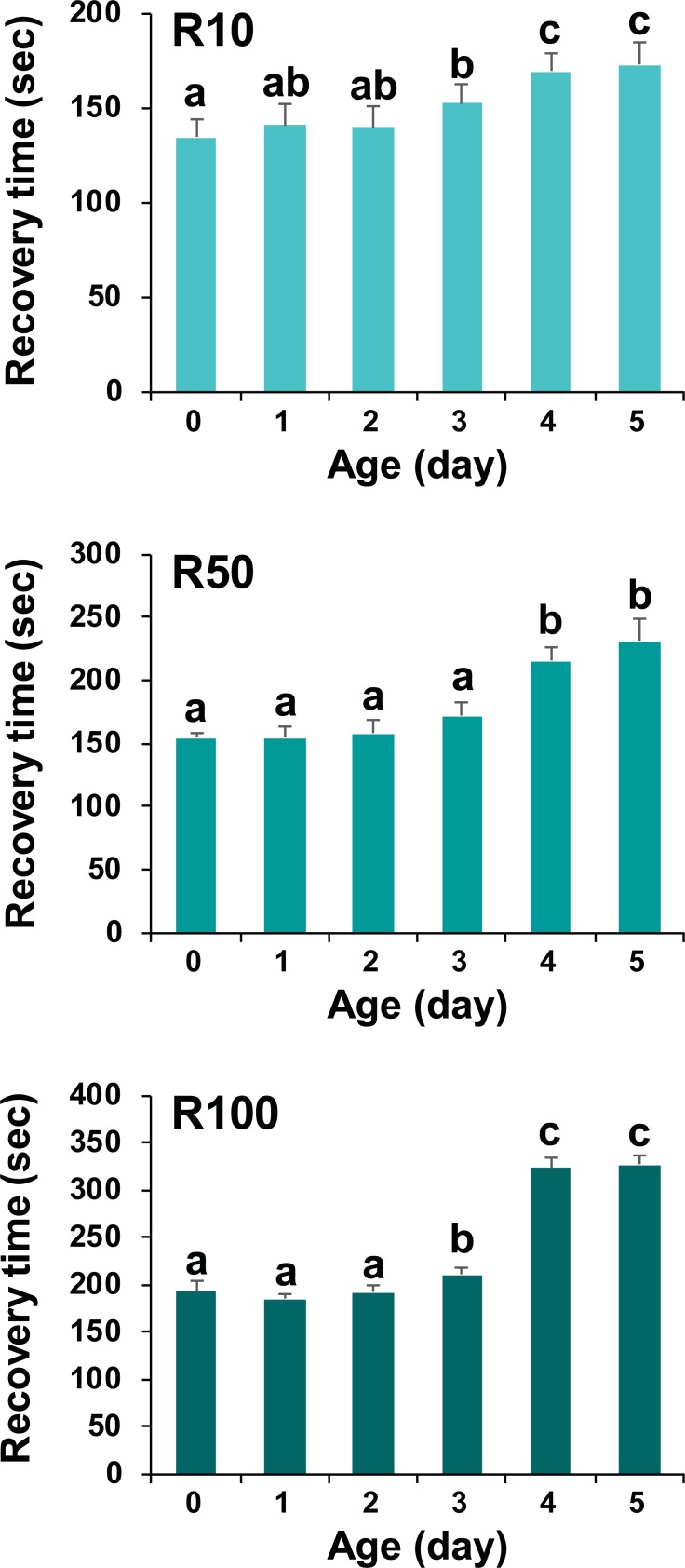
Effects of prolarval ages on recovery time after the anesthetic treatment with 200 mg/L of MS-222. Elapsed times to 10% (R10), 50% (R50) and 100% (R100) of fish (N = 20 per replicate; three replicates per age group) recovered were estimated with different age groups (Day 0 to Day 5). Mean ± SD with different letters (a-c) indicate the statistical difference at P < 0.05 based ANOVA followed by Tukey’s post hoc analysis. In contrast to recovery times, times to induction of anesthesia were not significantly influenced by ages of prolarvae (S5 Fig). Size of prolarvae and data on post-recovery viability are provided in S2 and S3 Tables, respectively. Age-dependent pattern of recovery time was also observable in prolarvae anesthetized with lidocaine (S6 Fig).

Anesthetic time with 200 mg/L of MS-222 was not significantly affected by prolarval ages. Prolarvae aged from 0–5 days displayed a similar time to anesthetic induction (S5 Fig). However, recovery time tended to increase with age. R10 recovery was similar among age groups from Day 0 to Day 2 (P > 0.05), but increased on Day 3 (P < 0.05). Subsequently, in Day 4 and Day 5 prolarvae, recovery times were significantly greater (P < 0.05). This pattern was similar for R50 and R100 recovery. Although the R50 recovery time of Day 3 prolarvae was similar to that of younger prolarvae, a lag in recovery was apparent in Day 4 and Day 5 groups (P < 0.05) (Fig 5). However, fish viability was not affected (S3 Table).

### Behavioral patterns of recovered larvae

**Fig 6:**
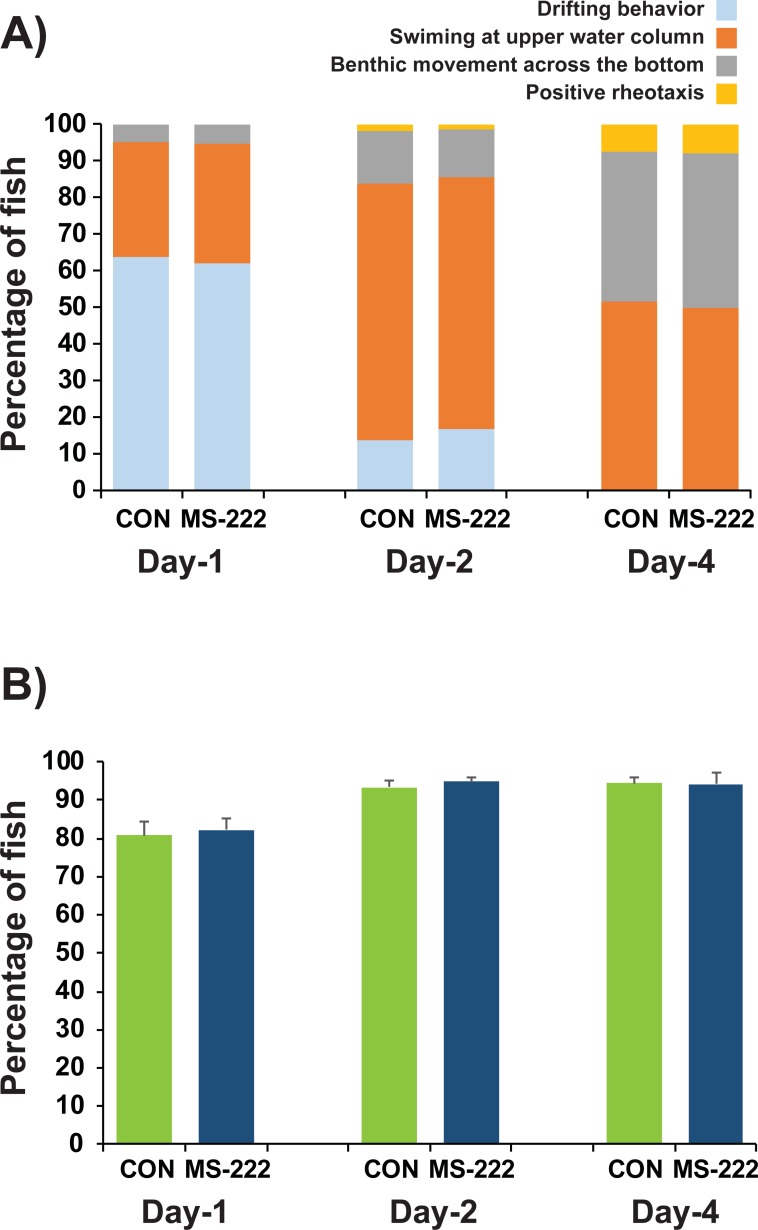
Post-recovery behavioral patterns of prolarvae after anesthetic treatment with 200 mg/L MS-222, compared with those of non-anesthetized control prolarvae. A) Swimming behaviors under daylight conditions. B) Phototactic response to a spotlight under dark conditions. No statistical difference was observed between MS-222-anesthetized and non-anesthetized prolarvae in all tests conducted. Triplicate examinations were made (N = 120 per replicate tank). Data in detail for each replicate tank under daylight and dark conditions are provided in S7 and S8 Figs, respectively. Data on comparison of post-recovery phototactic behavior after anesthetic treatment with MS-222 or lidocaine are provided in S9 Fig.

A. *baerii* prolarvae recovered from MS-222-anesthesia at 200 mg/L showed no apparent difference in the post-recovery behavioral characteristics compared to non-anesthetized fish (Experiment E). Under daylight conditions, prolarvae from different age groups (Day 1, Day 2, and Day 4) displayed ontogenetic behavioral patterns consistent with those described as normal for this sturgeon species (Fig 6A) [20]. Day 1 prolarvae showed characteristic behaviors of drifting and/or swimming in the upper water column. With age, the percentage of fish showing benthic movement across the tank bottom continually increased, whereas percentages of fish displaying drifting behavior and swimming at the water surface decreased. Of benthic swimmers, a small proportion of individuals showed positive rheotaxis on Day 4 (7–8%) (S7 Fig). Under dark conditions, *A*. *baerii* prolarvae showed a more or less uniform pattern of behavior irrespective of MS-222-anesthetized and control groups, characterized by benthic movement across the tank bottom. Age-dependent modifications of behavioral patterns were also not significantly different under dark conditions regardless of anesthesia (S8 Fig). In the assessment of phototaxis, both MS-222-anesthetized/recovered and control groups exhibited a similar degree of positive phototactic response to a spotlight under darkness at each age (Fig 6B) (P > 0.05).

### Microinjection of anesthetized prolarvae

**Fig 7:**
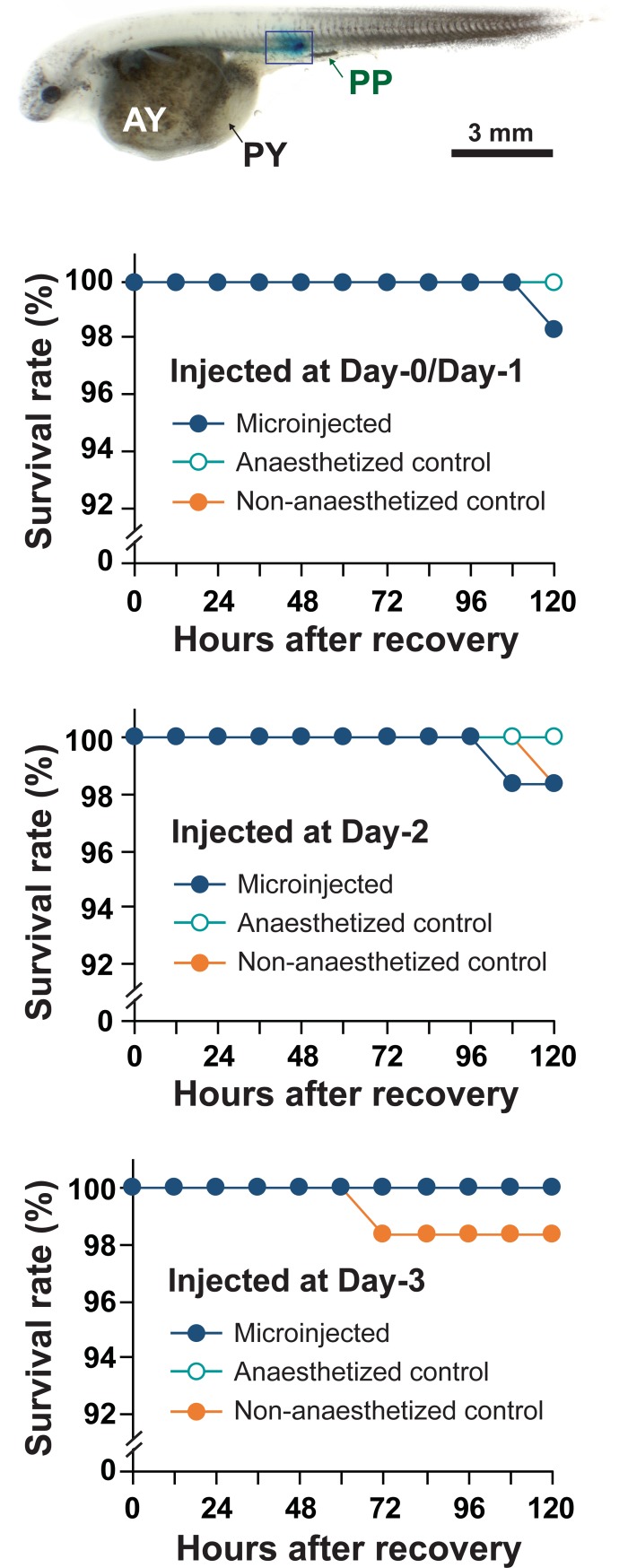
Demonstration of microinjection with anesthetized Siberian sturgeon prolarvae. Prolarvae at Day 0/Day1, Day 2, and Day 3 (N = 60 per day) were immobilized with MS-222 at 200 mg/L and microinjected with methylene blue dye at 0.5 mg/L. Post-operative survival for up to 120 h of injected prolarvae in comparison with groups receiving anesthesia but no injection or no anesthesia/no injection controls showed no significant difference among groups. A representative photograph of Day 3 prolarva showing the successful delivery of the dye through microinjection is also shown (AY, anterior part of yolk sac; PY, posterior part of yolk sac; and PP, pigment plug; injection site boxed).

We demonstrated the applicability of the present anesthetic protocol to the microinjection of *A*. *baerii* prolarvae. The concentration of methylene blue used in this study (0.5 mg/mL) did not influence the viability of *A*. *baerii* prolarvae injected. Delivery of dye into the border region between the prolarval body and the yolk extension was easily verified under the microinjection conditions used in this study. Of sixty prolarvae microinjected from each injection batch, the number of dead fish was zero or one until 120 h post injection, similar to both control groups (i.e., those anesthetized but not injected, and a non-anesthetized group) (Fig 7).

## Discussion

Based on a comparative examination on the potency of three selected anesthetics, MS-222 was proven to be the most efficient in inducing anesthesia of Siberian sturgeon prolarvae than clove oil or lidocaine. Clove oil was consistently shown to have slower induction times compared to lidocaine and MS-222. Although induction time of lidocaine was comparable to that of MS-222 at 100–200 mg/L doses (and was even shorter at the lowest dose of 50 mg/L), a dose-dependent decrease of the anesthetic time was not clearly achieved at the highest dose of 400 mg/L. The difference in the efficacy among anesthetics could be more clearly observed in the analysis of recovery times. Recovery of MS-222-treated prolarvae was obviously faster than those treated with clove oil or lidocaine at all the dose levels tested. Furthermore, interindividual variation in recovery time was significantly smaller in MS-222-anesthetized larvae, compared to those anesthetized with clove oil or lidocaine (Experiment A). Results from this study are in broad agreement with previous studies showing that MS-222 in an ideal anesthetic agent in fishes when rapid anesthesia and quick recovery are important [22]. MS-222 is a local anesthetic acting on voltage-sensitive sodium channels, and has been one of the most frequently used agents in various sturgeon species [23, 24, 25], although the anesthetic efficacy of MS-222 for yolk-bearing sturgeon larvae has thus far not been studied. Previously, dose levels of MS-222 used in different sturgeon species were suggested to be 80–250 mg/L with 4–15 min of induction time and 3–7 min of recovery time depending on species and developmental stages [26]. Although most previous studies have reported that MS-222 is an effective and safe anesthetic for juvenile and subadult sturgeon individuals, its use has not been recommended in certain sturgeon species such as the pallid sturgeon *Scaphirhynchus albus* due to potential mortality [27]. More recently, one study of Persian sturgeon (*Acipenser persicus*) 1-month-old fingerlings reported that concentrations of MS-222 ranging from 75 to 100 mg/L caused mortality [28], which is in contrast to our findings with Siberian sturgeon prolarvae. Hence, the adverse effects of MS-222 on viability of sturgeon may be species- and developmentally-specific. Despite the general agreement on the safety of MS-222, it has been reported to have adverse side effects such as aversion, epidermal and corneal lesions, hypoxemia, decreased heart rate, and mortality in some fish species depending on the dose and exposure times [2, 29, 30]. Although such concerns were not observed in this investigation, further studies are needed to test whether adverse effects of MS-222 appear to differ depending on the age or developmental stage of this sturgeon species.

Clove oil is believed to have a basic mechanism similar to MS-222, as a local or peripheral anesthetic and antinociceptive actor with its main action on specific voltage-gated sodium channels [31, 32]. Clove oil is actually a mixture of different compounds distilled from clove trees (*Eugenia caryophyllata* or *E*. *aromatica*), and eugenol [2 methoxy-4-(2-propenyl) phenol] is the principal active ingredient of clove oil. Due to its natural origin, low price, and ready availability, clove oil (or its main constituent, eugenol) is widely used as an anesthetic for fish species [1, 33, 34]. In Russian sturgeon juveniles, test concentrations of 450 to 900 mg/L were reported to meet the efficacy criteria specified for handling within 3 min, with recovery in 5 min and no mortality [35, 36]. Meanwhile, relatively higher doses of clove oil have been used to induce anesthesia of juveniles and subadults of white sturgeon (*Acipenser transmontanus*), with mean induction and recovery times for 1000 mg/L determined as 1.5 min and 16.3 min, respectively [37]. On the other hand, for Siberian sturgeon juveniles, substantially lower concentrations of clove oil (60 to 90 mg/L) were proposed as an appropriate dose range to minimize stress responses [38]. Another previous study on Siberian sturgeon fingerlings has indicated that the effective concentration of clove oil should be 330–381 mg/L [39]. However, in contrast to previous studies, our findings suggest that clove oil is not a suitable anesthetic for Siberian sturgeon prolarvae, due to prolonged recovery times (>8 min to >26 min with test doses 50 to 400 mg/L). A similar finding of slow induction and recovery times has been reported with isoeugenol-treated lumpfish [4]. Collectively, data from both previous and the present studies corroborates idea that the physiological effects of clove oil would be species-specific, and also suggests that that sturgeon species might show age (or size)-dependent responses to clove oil.

Lidocaine is also a sodium channel blocker and local anesthetic that has previously been used for anesthesia in medaka species (*Oryzias dancena*) with large margin of safety [40]. This chemical has been considered an alternative candidate to MS-222 based on its low cost and ease of preparation. In zebrafish (*Danio rerio*), lidocaine-HCl has been claimed to have advantageous merits over MS-222, such as longer duration of surgical anesthesia, less adverse effects on heart rate, and more complete euthanasia [41]. This anesthetic has also been used as combined with other anesthetics (e.g., propofol) in zebrafish to overcome its potential drawbacks such as high mortality rates at high concentrations and high variability in anesthetic/recovery times [2, 22]. For sturgeons, lidocaine has been little used and must be assessed according to the criteria of efficacy evaluation for induced anesthesia. From this study, the potency of lidocaine to induce anesthesia of sturgeon prolarvae was similar to MS-222, and lidocaine did not cause significant mortality. However, the recovery of lidocaine-treated prolarvae was not as efficient as MS-222-treated fish.

Based on our assessment of individual variations in anesthetic induction and recovery times with test concentrations 50, 100, 200, and 400 mg/L of MS-222 (Experiment B), the lowest concentration of MS-222 showed large interindividual variations in both induction and recovery times, while the highest concentration was proven to give rise to a small portion of individuals requiring prolonged recovery, which might be undesirable for the handling of a large number of prolarvae for microinjection. Hence, from the viewpoint of uniformity in induction and recovery times, an appropriate dosage of MS-222 for Siberian sturgeon prolarvae could be 100 to 200 mg/L.

However, given the purpose of microinjection, another important requirement of a suitable anesthetic protocol is the ability to stably maintain a deep anesthetic state for a sufficient period of time. The microinjection procedure includes arranging anesthetized larvae, injection to the target site of each individual, then transferring the injected individuals to a recovery tank [7, 10]. During this practice, larvae are usually exposed to the air rather than immersed in water, and their reflex activity should be firmly inhibited (i.e., immobilization in an unconscious state). Under these criteria, our findings in Experiment C comparing reflex activity clearly recommended a dose of 200 mg/L rather than 100 mg/L, since a considerable portion of 100 mg/L MS-222-anesthetized larvae did not remain in an unconscious state after 2 min of air exposure. Because the larvae underwent an additional 5 min of air exposure after reaching anesthesia, recovery times increased in both 100 mg/L and 200 mg/L concentrations compared to the recovery by direct transfer of larvae to clean water immediately after reaching anesthesia. Nevertheless, the increased anesthetic duration in the air did not cause any adverse effect on the post-recovery survival of the prolarvae.

Based on the results of Experiment D, prolarval age significantly influenced recovery time, where older prolarvae (>Day 3) required a longer time to recover than did younger individuals, although there was no effect on the induction time of anesthesia. Although we have not yet clarified the mechanism responsible for this observation, one plausible explanation is that it could be related to ontogenetic difference in gill development. Post-larval fish (and fish at subsequent stages of development) use gills as the main organ for oxygen uptake, respiratory gas exchange, and other physiologic functions; hence, the gill may be the main route of anesthetic uptake during immersion treatment [4, 42]. However, newly hatched and early prolarvae with poorly developed or undeveloped gills may use the skin more than gills for respiratory exchange and other relevant functions, and may therefore show different responses to immersion exposure to anesthetics compared to older fishes with well-developed gills [42, 43]. The remarkable difference in the response to anesthetic treatments between larvae and adults has been previously highlighted in zebrafish [44]. Sturgeons are known to possess external gills that are not completely covered by an operculum in their early life stages [18]. Newly hatched prolarvae (Day 0) of Siberian sturgeon display only brachial grooves, without clear development of defined gill filaments. This rudimentary structure is usually seen until Day 1. On Day 2, early development of external gills become visible; after this, the external gills are vigorously developed in subsequent ages (S10 Fig). [19]. With this ontogenetic viewpoint, the delay of recovery time in aged prolarvae might be, at least in part, related to the delivery of higher amounts of dissolved anesthetic compounds through the developed external gills in older prolarvae compared to younger ones. However, despite delayed recovery times, the post-recovery viability of prolarvae was not affected by age, suggesting good safety margins regardless of larval age.

Based on our assessment of post anesthetic/recovery behaviors (Experiment E), the prolarvae quickly resumed normal swimming behaviors comparable to those observed in the non-anesthetized control prolarvae. Siberian sturgeons are known to undergo a dynamic change in behavioral patterns, with these including the progressive disappearance of drifting behavior, the transition from pelagic swimming in upper water column to benthic swimming, and the acquisition of strong positive phototaxis [45]. These behaviors were observed to take place during the early prolarval period (up to Day 4 in this study), and were generally in accordance with previous reports [20, 21]. Furthermore, our data indicated clearly that treatment with MS-222 did not give rise to any effects on the behavior of recovered prolarvae. Meanwhile, local anesthetics including MS-222 could potentially influence the sensitivity of the nociceptors to stimulation [3, 32]. However, testing of post-exposure analgesic efficacy (e.g., noxious stimulus using acetic acid pain test) has not yet been conducted with Siberian sturgeon prolarvae, and should be carried out in future studies.

Finally, we verified the applicability of the recommended anesthetic protocol to microinjection-related handling. With prolarval specimens anesthetized with MS-222, routine microinjection practice could be feasible without any impaired viability of fish, based on our observation of very low post-operative mortality. No apparent difference in post-recovery survival rate was detected among prolarvae subjected to microinjection, anesthesia only, or non-anesthesia control. Taken together, the results of this study suggest that an anesthetic protocol based on 200 mg/L of MS-222 could provide researchers with a practical approach for the micromanipulation and other related handling of Siberian sturgeon prolarvae, as evidenced by rapid induction of anesthesia, stable inhibition of reflex activity for a sufficient period for handling, fast and uniform recovery time, and good safety margins.

## Supporting information

S1 Fig Anesthetic induction of *A*. *baerii* prolarvae treated with MS-222 (200 mg/L) or lidocaine (200–1600 mg/L). Triplicate examinations were made (N = 20 per replicate treatment). Mean ± SD with different letters (a-b) indicate the statistical difference at P < 0.05 based ANOVA followed by Tukey’s post hoc test.
(PDF)

S2 Fig Recovery times after anesthetic immersion treatment of newly hatched *A*. *baerii* prolarvae with clove oil, lidocaine, and MS-222. For each anesthetic agent, four different doses (50, 100, 200, and 400 mg/L) were tested and times for 10% (R10), 50% (R50), and 100% (R100) of prolarvae recovered were determined. With a given anesthetic agent, means ± SD with different letters (α-δ, a-d, or w-z) were significantly different based on ANOVA followed by Tukey’s post hoc analysis at P < 0.05.
(PDF)

S3 Fig Recovery times of *A*. *baerii* prolarvae after anesthetic treatment with MS-222 (200 mg/L) or lidocaine (200–1600 mg/L). Mean ± SD with different letters (a-e) indicate the statistical difference at P < 0.05 based ANOVA followed by Tukey’s post hoc test. Numerical value in percentage above each histogram is post-anesthesia survival (mean ± SD).
(PDF)

S4 Fig Comparison of reflex activity against external stimuli of *A*. *baerii* prolarvae treated with MS-222 (200 mg/L) or lidocaine (200 mg/L). (A) Proportion (%) of prolarvae showing no reflex response. (B) Recovery times after anesthesia followed by the exposure to room air for 5 min. (C) Post-anesthesia survival rates.
(PDF)

S5 Fig Effect of prolarval ages on anesthetic induction times during immersion treatment with MS-222 (200 mg/L). No significant difference was found among age groups.
(PDF)

S6 Fig Effects of prolarval ages (Day 0 and Day 5) on recovery time after anesthetic treatment with MS-222 (200 mg/L) or lidocaine (200 mg/L). Similar with MS-222, older prolarvae showed a longer recovery time than did younger fish after anesthetic treatment with lidocaine based on student’s *t-*test at P < 0.05.
(PDF)

S7 Fig Swimming and behavioral patterns of prolarvae after recovery from the anesthesia with MS-222 (200 mg/L) under daylight conditions. Post-recovery behaviors were compared with those of non-anesthetized control prolarvae under daylight conditions based on three replicate tanks at Day 1, Day 2, and Day 3 ages. No apparent difference was found between anesthetized and non-anesthetized groups, irrespective of age. Behavioral criteria were made according to a previous study [20].
(PDF)

S8 Fig Swimming and behavioral patterns of prolarvae after recovery from anesthesia with MS-222 (200 mg/L) under dark conditions. Most prolarvae of Siberian sturgeon species display active movement across the bottom of the tank in darkness. No apparent difference was found between anesthetized and non-anesthetized groups, irrespective of age.
(PDF)

S9 Fig Post-recovery phototaxis behavior of prolarvae after anesthetic treatment with MS-222 (200 mg/L) or lidocaine (200 mg/L). Positive phototactic behavior was examined in response to a spotlight under dark conditions on Day 2 and Day 4. There was no significant difference in phototactic characteristics of prolarvae anesthetized with MS-222 or lidocaine based on ANOVA and/or student’s *t*-test (P > 0.05).
(PDF)

S10 Fig Representative photographs to show the development of external gills in *Acipenser baerii* during the prolarval stage from Day 0 to Day 5. Abbreviations are barbel rudiment (BR), brachial grooves (BG), external gills (EXG), myelencephalon cavity (MyC), olfactory pit (OP), and yolk sac (YS). Bars indicate 0.3 mm.
(PDF)

S1 Table Quality parameters of water used for anesthetic/recovery experiments in this study. (PDF)

S2 Table Average total length and body weight of Siberian sturgeon *A*. *baerii* prolarvae during the ages Day 0 to Day 8. (PDF)

S3 Table Post-anesthesia/recovery viability of *A*. *baerii* prolarvae assessed in this study. (PDF)

## References

[1] Zahl IH, Samuelsen O, Kiessling A. Anaesthesia of farmed fish: implications for welfare. Fish Physiol Biochem. 2012; 38:201–18. 10.1007/s10695-011-9565-1 22160749

[2] Martins T, Diniz E, Félix LM, Antunes L. Evaluation of anaesthetic protocols for laboratory adult zebrafish (*Danio rerio*). PLoS One. 2018; 13(5):e0197846 10.1371/journal.pone.0197846 29787611 PMC5963751

[3] Nordgreen J, Tahamtani FM, Janczak AM, Horsberg TE. Behavioural effects of the commonly used fish anaesthetic tricaine methanesulfonate (MS-222) on zebrafish (*Danio rerio*) and its relevance for the acetic acid pain test. PLoS One. 2014; 9(3):e92116 10.1371/journal.pone.0092116 24658262 PMC3962382

[4] Skår MW, Haugland GT, Powell MD, Wergeland HI, Samuelsen OB. Development of anaesthetic protocols for lumpfish (*Cyclopterus lumpus* L.): Effect of anaesthetic concentrations, sea water temperature and body weight. PLoS One. 2017; 12(7):e0179344 10.1371/journal.pone.0179344 28678815 PMC5497946

[5] Samaee S, Nikkhah H, Varga AM, Rezaei B. A simple and inexpensive microinjection system for zebrafish embryos and larvae. Zebrafish. 2017; 14(6):581–5. 10.1089/zeb.2017.1425 28678656

[6] Astin JW, Keerthisinghe P, Du L, Sanderson LE, Crosier KE, Crosier PS et al Innate immune cells and bacterial infection in zebrafish. Methods Cell Biol. 2017; 138:31–60. 10.1016/bs.mcb.2016.08.002 28129850

[7] Robles V, Riesco MF, Psenicka M, Saito T, Valcarce DG, Cabrita E et al Biology of teleost primordial germ cells (PGCs) and spermatogonia: Biotechnological applications. Aquaculture. 2017; 472:4–20. 10.1016/j.aquaculture.2016.03.004

[8] Tonelli FMP, Lacerda SMSN, Tonelli FCP, Costa GMJ, França LR, Resende RR. Progress and biotechnological prospects in fish transgenesis. Biotechnol Adv. 2017; 35:832–44. 10.1016/j.biotechadv.2017.06.002. 28602961

[9] Rønnestad I, Dominguez RP, Tanaka M. Ontogeny of digestive tract functionality in Japanese flounder, *Paralichthys olivaceus* studied by *in vivo* microinjection: pH and assimilation of free amino acids. Fish Physiol Biochm. 2000; 22:225–35. 10.1023/A:1007801510056

[10] Ellett F, Irimia D. Microstructured surface arrays for injection of zebrafish larvae. Zebrafish. 2017; 14(2):140–5. 10.1089/zeb.2016.1402 28151697 PMC5385424

[11] Kim DS, Nam YK, Noh JK, Park CH, Chapman FA. Karyotype of North American shortnose sturgeon *Acipenser brevirostrum* with the highest chromosome number in the Acipenseriformes. Ichthyol Res. 2005; 52: 94–7. 10.1007/s10228-004-0257-z

[12] Trifonov VA, Romanenko SS, Beklemisheva VR, Biltueva LS, Makunin AI, Lemskaya NA et al Evolutionary plasticity of acipenseriform genomes. Chromosoma. 2016; 125(4):661–8. 10.1007/s00412-016-0609-2 27411693

[13] Cho YS, Douglas SE, Gallant JW, Kim KY, Kim DS, Nam YK. Isolation and characterization of cDNA sequences of L-gulono-gamma-lactone oxidase, a key enzyme for biosynthesis of ascorbic acid, from extant primitive fish groups. Comp Biochem Physiol. B. 2007; 147: 178–90. 10.1016/j.cbpb.2007.01.001 17317254

[14] Pšenička M, Saito T, Linhartová Z, Gazo I. Isolation and transplantation of sturgeon early-stage germ cells. Theriogenology. 2015; 83: 1085–92. 10.1016/j.theriogenology.2014.12.010 25559841

[15] Saito T, Pšenička M. Novel Technique for visualizing primordial germ cells in sturgeons (*Acipenser ruthenus*, *A*. *gueldenstaedtii*, *A*. *baerii*, and *Huso huso*). Biol Repro. 2015; 93(4):96, 1–7. 10.1095/biolreprod.115.128314 26134864

[16] Webb MAH, Doroshov SI. Importance of environmental endocrinology in fisheries management and aquaculture of sturgeons. Gen Comp Endocrinol. 2011; 170:313–21. 10.1016/j.ygcen.2010.11.024 21130093

[17] Park CH, Lee SY, Kim DS, Nam YK. Embryonic development of Siberian sturgeon *Acipenser baerii* under hatchery conditions: an image guide with embryological descriptions. Fish Aquat Sci. 2013; 16:15–23. 10.5657/fas.2013.0015

[18] Park CH, Lee SY, Kim DS, Nam YK. Effects of incubation temperature on egg development, hatching and pigment plug evacuation in farmed Siberian sturgeon *Acipenser baerii*. Fish Aquat Sci. 2013; 16:25–34. 10.5657/fas.2013.0025

[19] Gisbert E, Nam YK. Early Ontogeny in the Siberian Sturgeon In: Williot P, Nonnotte G, Vizziano-Cantonnet D, Chebanov M, editors. The Siberian Sturgeon (*Acipenser baerii*, Brandt, 1869). Springer International Publishing; 2018 Volume 1 –Biology. pp. 131–57.

[20] Gisbert E, Williot P, Castelló‐Orvay F. Behavioural modifications in the early life stages of Siberian sturgeon (*Acipenser baerii*, Brandt). J Appl Ichthyol. 1999; 15(4‐5), 237–42. 10.1111/j.1439-0426.1999.tb00242.x

[21] Gisbert E, Ruban GI. Ontogenetic behavior of Siberian sturgeon, *Acipenser baerii*: A synthesis between laboratory tests and field data. Environ Biol Fish. 2003; 67(3), 311–9. 10.1023/a:1025851502232

[22] Valentim AM, Félix LM, Carvalho L, Diniz E, Antunes LM. A new anaesthetic protocol for adult zebrafish (*Danio rerio*): propofol combined with lidocaine. PLoS One. 2016; 11(1):e0147747 10.1371/journal.pone.0147747 26808508 PMC4725851

[23] Gomulka P, Wlasow T, Velíšek J, Svobodová Z, Chmielinska E. Effects of eugenol and MS-222 anaesthesia on Siberian sturgeon *Acipenser baerii* Brandt. Acta Vet Brno. 2008; 77:447–53. 10.2754/avb200877030447

[24] Di Marco, Petochi T, Longobardi A, Priori A, Finoia MG, Donadelli V et al Efficacy of tricaine methanesulphonate, clove oil and medetomidine-ketamine and their side effects on the physiology of sturgeon hybrid *Acipenser naccarii* x *Acipenser baerii*. J Appl Ichthyol. 2011; 27:611–7. 10.1111/j.1439-0426.2011.01701.x

[25] Matsche MA. Evaluation of tricaine methanesulfonate (MS-222) as a surgical anesthetic for Atlantic sturgeon *Acipenser oxyrinchus oxyrinchus*. J Appl Ichthyol. 2011; 27:600–10. 10.1111/j.1439-0426.2011.01714.x

[26] Popovic NT, Strunjak-Perovic I, Coz-Rakovac R, Barisic J, Jadan M, Berakovic AP et al Tricaine methane-sulfonate (MS-222) application in fish anaesthesia. J. Appl. Ichthyol. 2012; 28(4):553–64. 10.1111/j.1439-0426.2012.01950.x

[27] Wanner GA. Evaluation of a gastric lavage method on juvenile pallid sturgeon. N Am J Fish Manage. 2006; 26:587–91. 10.1577/M05-090.1

[28] Falahatkar B, Poursaeid S. Anaesthetic efficacy of tricaine methanesulfonate for Persian sturgeon larvae. Aquac Res. 2017; 48:4578–81. 10.1111/are.13084

[29] Huang WC, Hsieh YS, Chen IH, Wang CH, Chang HW, Yang CC, et al Combined use of MS-222 (tricaine) and isoflurane extends anesthesia time and minimizes cardiac rhythm side effects in adult zebrafish. Zebrafish. 2010; 7(3):297–304. 10.1089/zeb.2010.0653 20807039

[30] Readman GD, Owen SF, Murrell JC, Knowles TG. Do fish perceive anaesthetics as aversive? PLoS One. 2013; 8(9):e73773 10.1371/journal.pone.0073773 24086294 PMC3781131

[31] Markowitz K, Moynihan M, Liu M, Kim S. Biologic properties of eugenol and zinc oxide-eugenol: a clinically oriented review. Oral Surg Oral Med Oral Pathol. 1992: 73:729–37. 10.1016/0030-4220(92)90020-Q 1437045

[32] Baldisserotto B, Parodi TV, Stevens ED. Lack of postexposure analgesic efficacy of low concentrations of eugenol in zebrafish, Vet Anaesth Analg. 2018; 45:48–56. 10.1016/j.vaa.2017.08.009 29239756

[33] Roubach R, Gomes LC, Fonseca FAL, Val AL. Eugenol as an efficacious anaesthetic for tambaqui, Colossoma macropomum (Cuvier). Aquac Res. 2005; 36:1056–61. 10.1111/j.1365-2109.2005.01319.x

[34] Hoseini SM, Rajabiesterabadi H, Tarkhani R. Anaesthetic efficacy of eugenol on iridescent shark, *Pangasius hypophthalmus* (Sauvage, 1878) in different size classes. Aquac Res. 2015; 46:405–12. 10.1111/are.12188

[35] Marking LL, Meyer FP. Are better anesthetics needed in fisheries? Fisheries. 1985; 10(6):2–5. 10.1577/1548-8446(1985)010<0002:ABANIF>2.0.CO;2

[36] Akbulut B, Çakmak E, Aksungur N, Çavdar Y. Effect of exposure duration on time to recovery from anaesthesia of clove oil in juvenile of Russian sturgeon. Turk J Fish Aquat Sci. 2011; 11:463–7. 10.4194/1303-2712-v11_3_17

[37] Taylor PW, Roberts SD. Clove oil: an alternative anaesthetic for aquaculture. N Am J Aquac. 1999; 61:150–5. 10.1577/1548-8454(1999)061<0150:COAAAF>2.0.CO;2

[38] Feng G, Zhuang P, Zhang L, Kynard B, Shi X, Duan M et al Effect of anaesthetics MS-222 and clove oil on blood biochemical parameters of juvenile Siberian sturgeon (*Acipenser baerii*). J. Appl. Ichthyol. 2011; 27:595–9. 10.1111/j.1439-0426.2011.01711.x

[39] Akbulut B, Çakmak E, Aksungur N, Çavdar Y. Effect of anaesthesia with clove oil and benzocaine on feed intake in Siberian sturgeon (*Acipenser baerii* Brandt, 1869). Turk J Fish Aquat Sci. 2012; 12:667–73. 10.4194/1303-2712-v12_3_15

[40] Park IS, Park SJ, Gil HW1, Nam YK, Kim DS. Anesthetic effects of clove oil and lidocaine-HCl on marine medaka (*Oryzias dancena*). Lab Animal. 2011; 40(2):45–51. 10.1038/laban0211-45 21252980

[41] Collymore C, Tolwani A, Lieggi C, Rasmussen S. Efficacy and safety of 5 anesthetics in adult zebrafish (*Danio rerio*). J Am Assoc Lab Anim Sci. 2014; 53(2):198–203. 24602548 PMC3966278

[42] Rombough PJ. Ontogenetic changes in the toxicity and efficacy of the anaesthetic MS222 (tricaine methanesulfonate) in zebrafish (*Danio rerio*) larvae. Comp Biochem Physiol A. 2007; 148:463–9. 10.1016/j.cbpa.2007.06.415 17643329

[43] Strykowski JL, Schech JM. Effectiveness of recommended euthanasia methods in larval zebrafish (*Danio rerio*). J Am Assoc Lab Anim Sci 2015; 54:81–84. 25651096 PMC4311746

[44] Collymore C, Banks EK, Turner PV. Lidocaine hydrochloride compared with MS222 for the euthanasia of zebrafish (*Danio rerio*). J Am Assoc Lab Anim Sci. 2016; 55(6):816–20. 27931323 PMC5113886

[45] Gisbert E, Solovyev M. Behaviour of early life stages in the Siberian sturgeon In: Williot P, Nonnotte G, Vizziano-Cantonnet D, Chebanov M, editors. The Siberian Sturgeon (*Acipenser baerii*, Brandt, 1869). Springer International Publishing; 2018 Volume 1 –Biology. pp. 159–72.

