# Supplementary figures and images for: Anesthetic protocol for microinjection-related handling of Siberian sturgeon (Acipenser baerii; Acipenseriformes) prolarvae
Source: PLoS One. 2018 Dec 31;13(12):e0209928. doi: 10.1371/journal.pone.0209928 (PMC6312391; doi:10.1371/journal.pone.0209928)

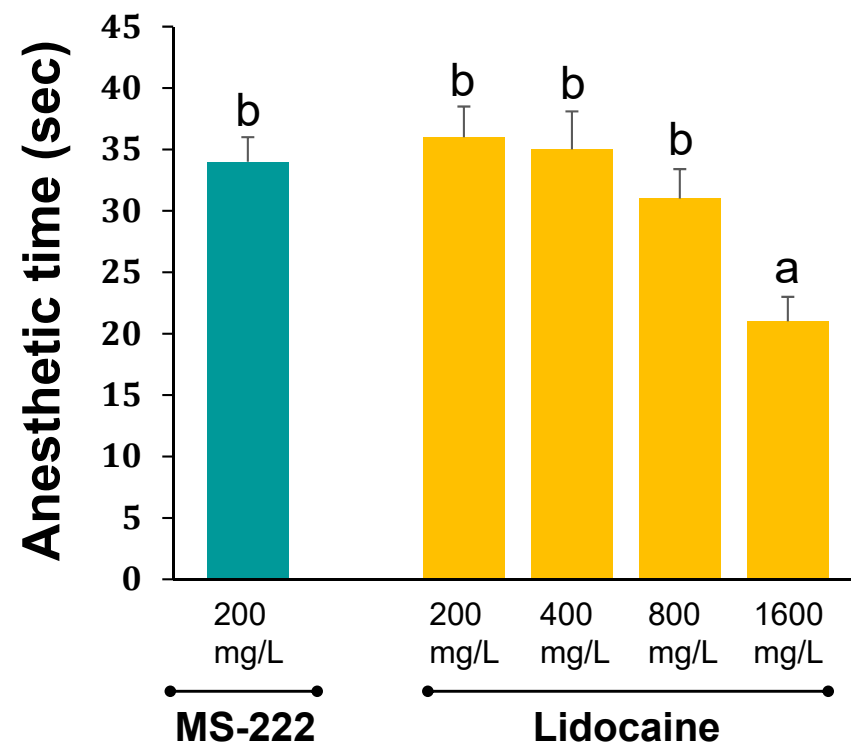

Supplement: S1 Fig — Triplicate examinations were made (N = 20 per replicate treatment). Mean ± SD with different letters (a-b) indicate the statistical difference at P < 0.05 based ANOVA followed by Tukey’s post hoc test. (PDF) [file pone.0209928.s001.pdf]

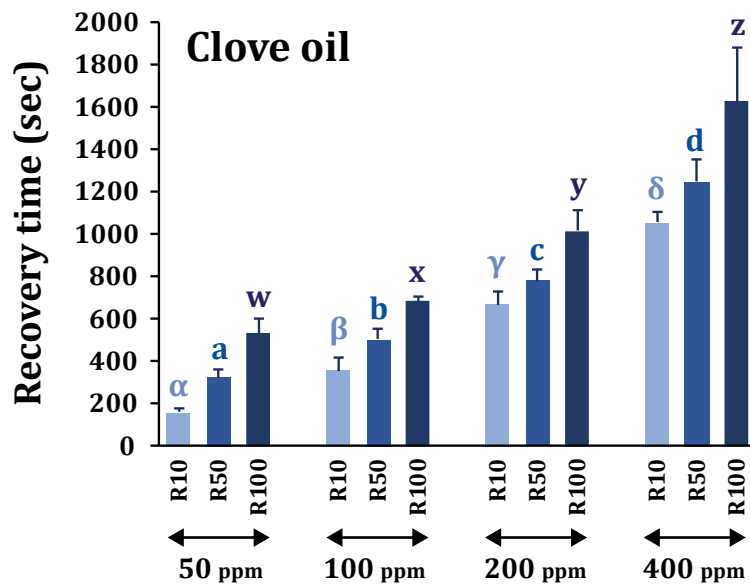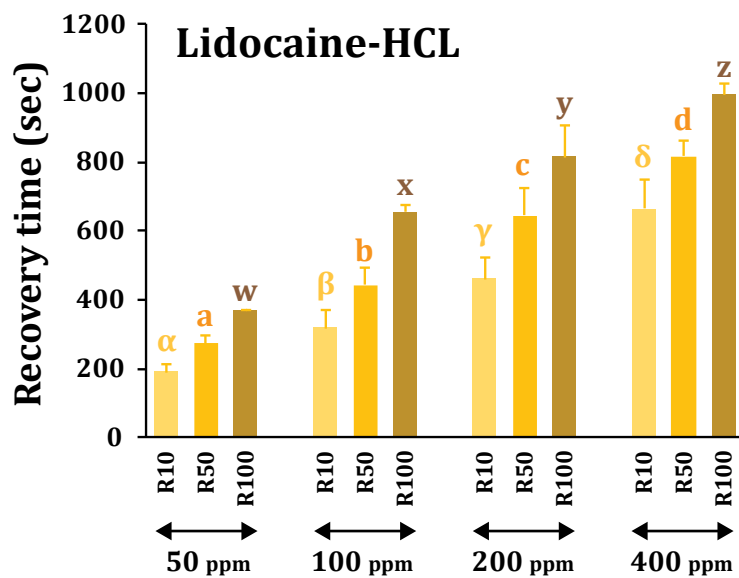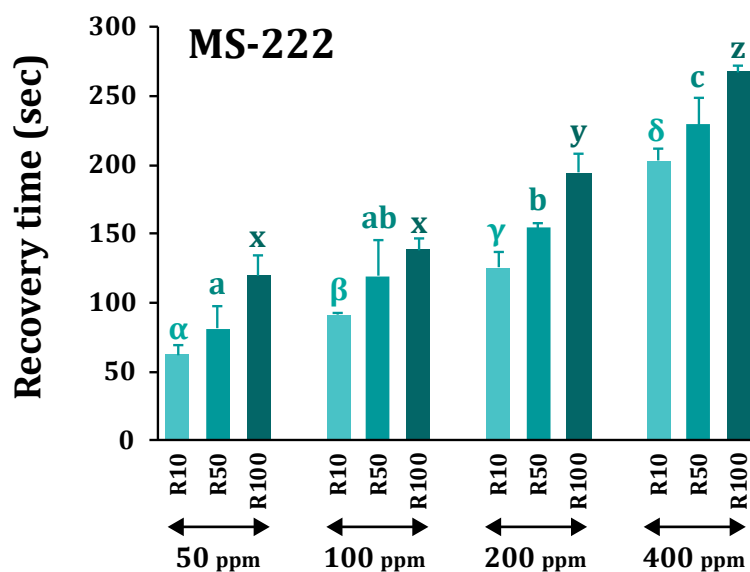

Supplement: S2 Fig — For each anesthetic agent, four different doses (50, 100, 200, and 400 mg/L) were tested and times for 10% (R10), 50% (R50), and 100% (R100) of prolarvae recovered were determined. With a given anesthetic agent, means ± SD with different letters (α-δ, a-d, or w-z) were significantly different based on ANOVA followed by Tukey’s post hoc analysis at P < 0.05. (PDF) [file pone.0209928.s002.pdf]

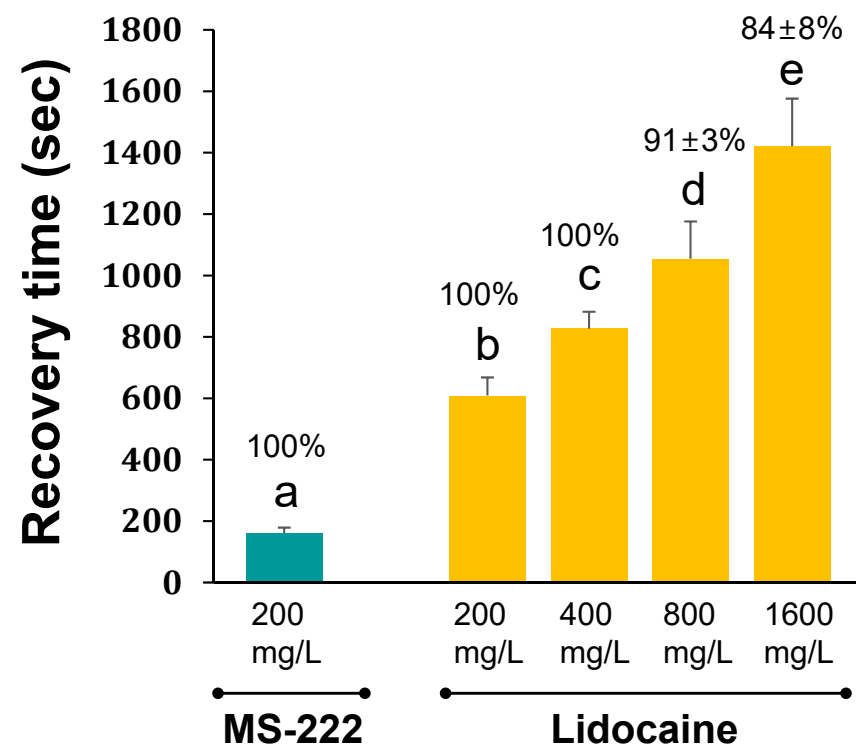

Supplement: S3 Fig — Mean ± SD with different letters (a-e) indicate the statistical difference at P < 0.05 based ANOVA followed by Tukey’s post hoc test. Numerical value in percentage above each histogram is post-anesthesia survival (mean ± SD). (PDF) [file pone.0209928.s003.pdf]

**A)**

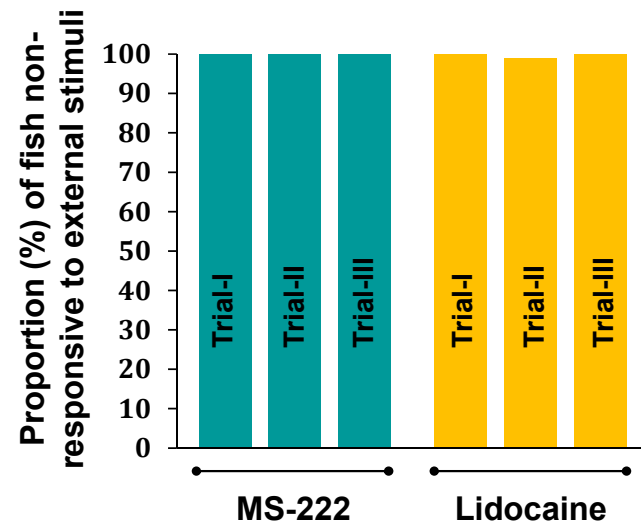

**B)**

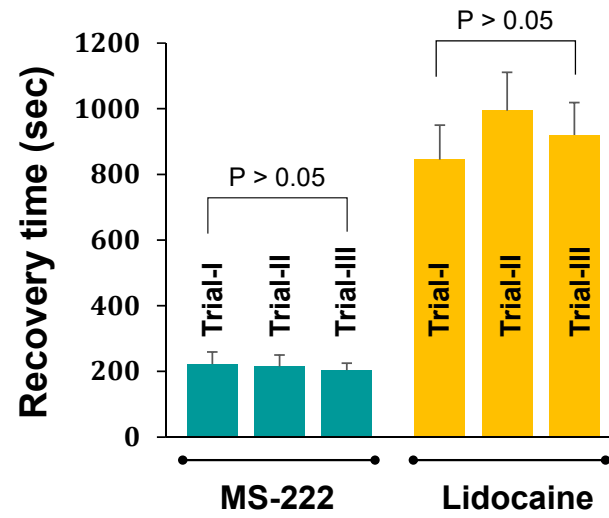

**C)**

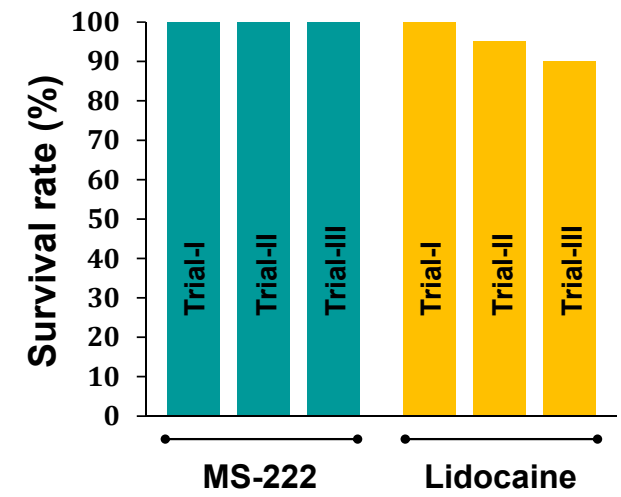

Supplement: S4 Fig — (A) Proportion (%) of prolarvae showing no reflex response. (B) Recovery times after anesthesia followed by the exposure to room air for 5 min. (C) Post-anesthesia survival rates. (PDF) [file pone.0209928.s004.pdf]

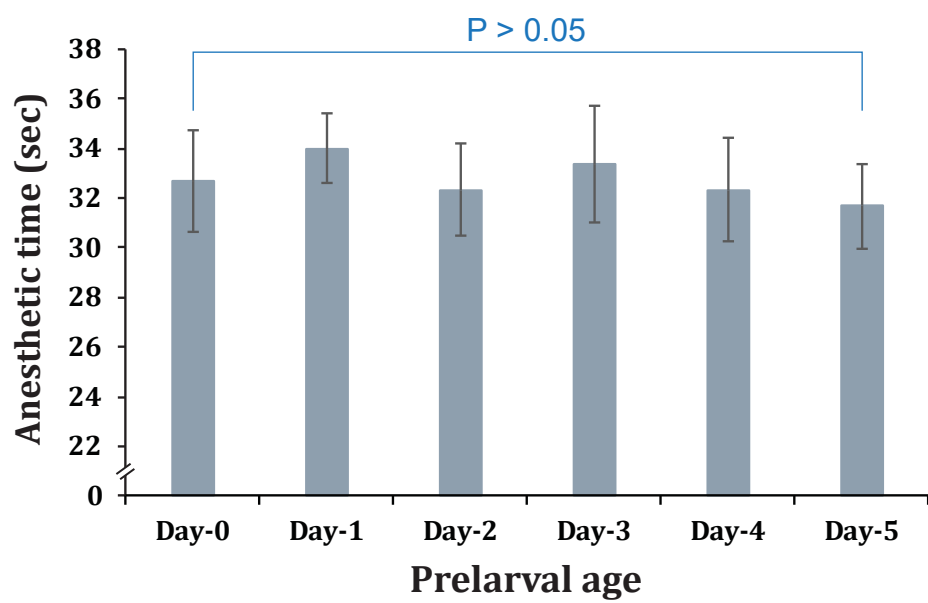

Supplement: S5 Fig — No significant difference was found among age groups. (PDF) [file pone.0209928.s005.pdf]

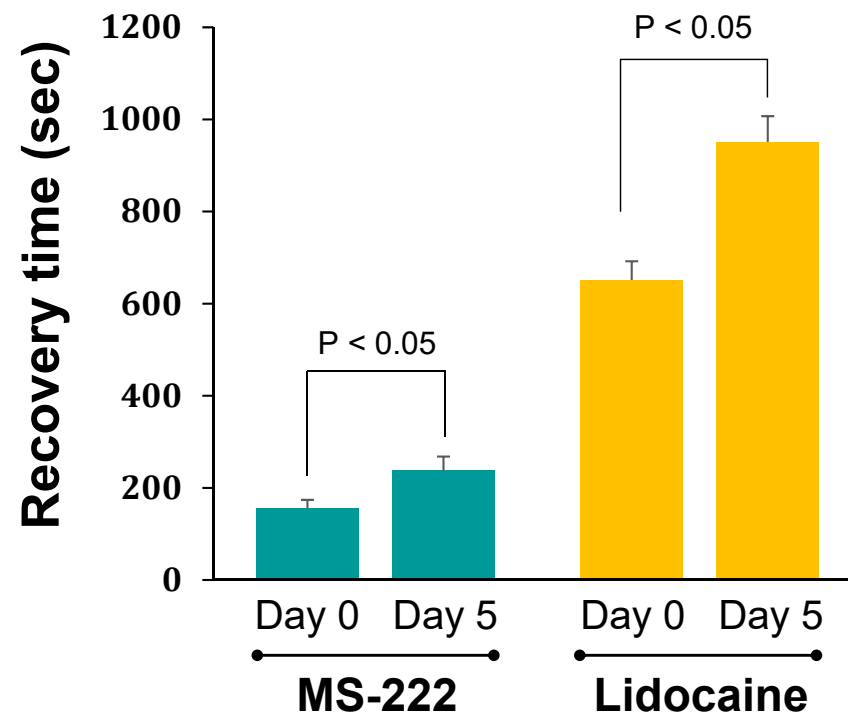

Supplement: S6 Fig — Similar with MS-222, older prolarvae showed a longer recovery time than did younger fish after anesthetic treatment with lidocaine based on student’s t-test at P < 0.05. (PDF) [file pone.0209928.s006.pdf]

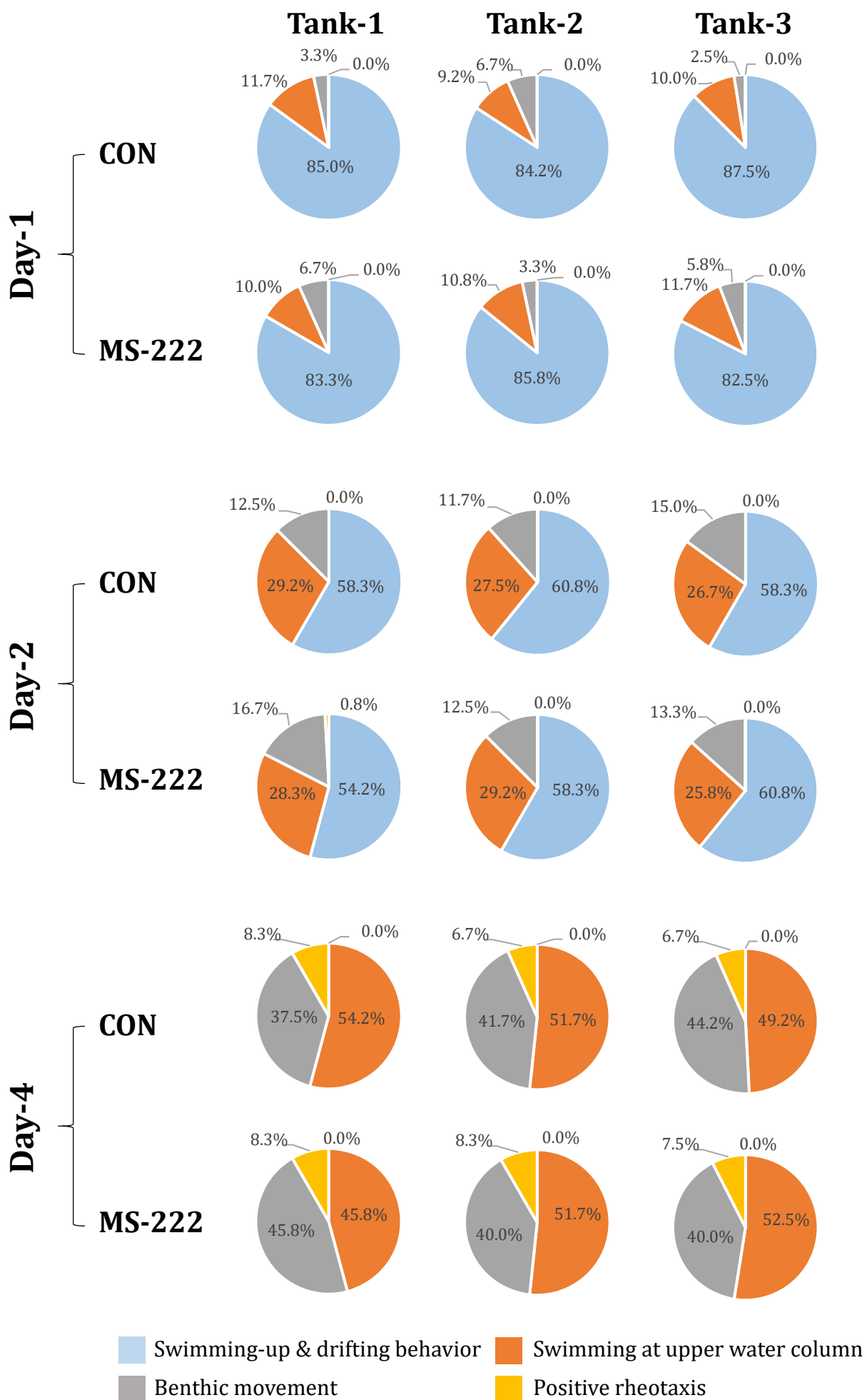

Supplement: S7 Fig — Post-recovery behaviors were compared with those of non-anesthetized control prolarvae under daylight conditions based on three replicate tanks at Day 1, Day 2, and Day 3 ages. No apparent difference was found between anesthetized and non-anesthetized groups, irrespective of age. Behavioral criteria were made according to a previous study [20]. (PDF) [file pone.0209928.s007.pdf]

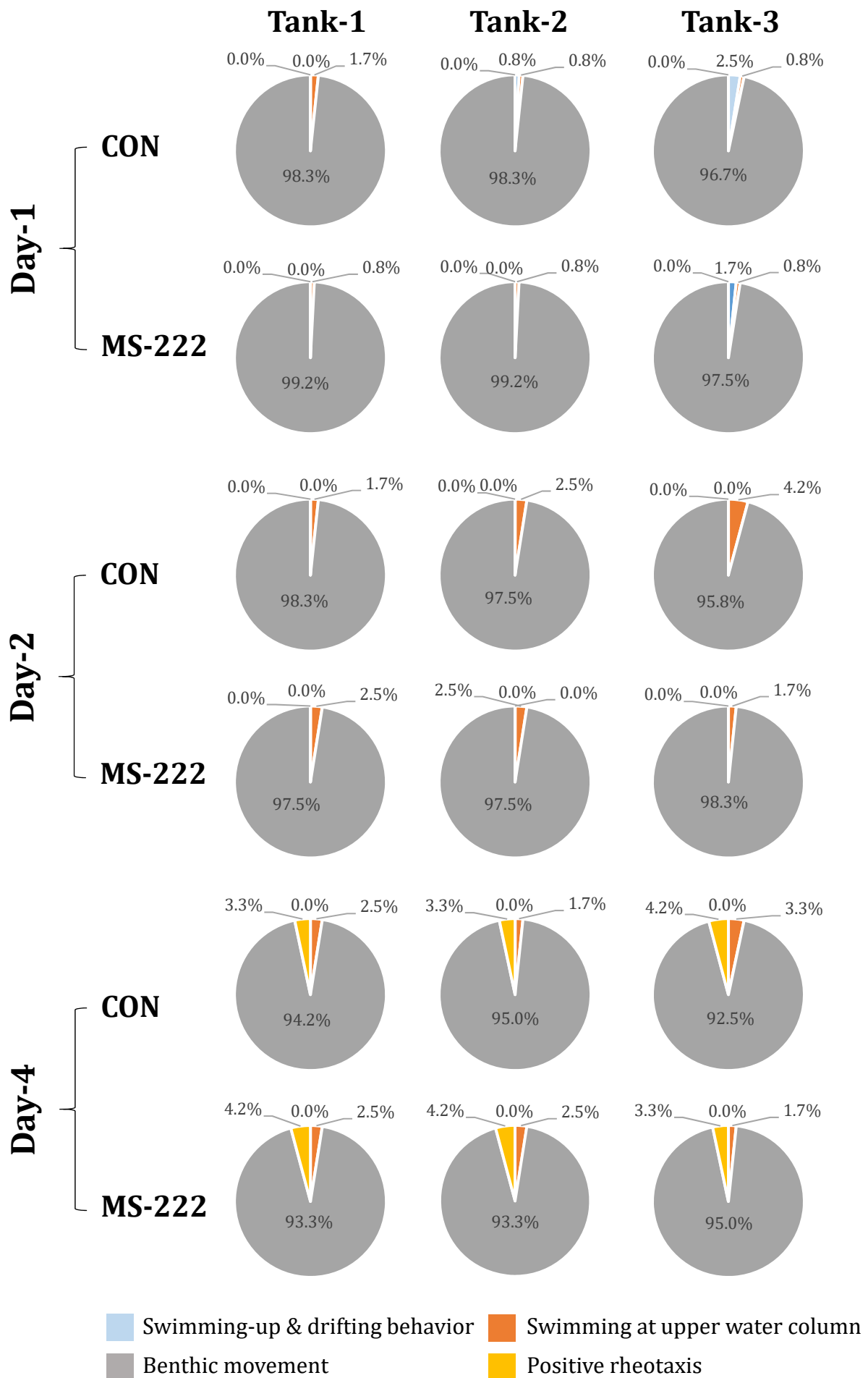

Supplement: S8 Fig — Most prolarvae of Siberian sturgeon species display active movement across the bottom of the tank in darkness. No apparent difference was found between anesthetized and non-anesthetized groups, irrespective of age. (PDF) [file pone.0209928.s008.pdf]

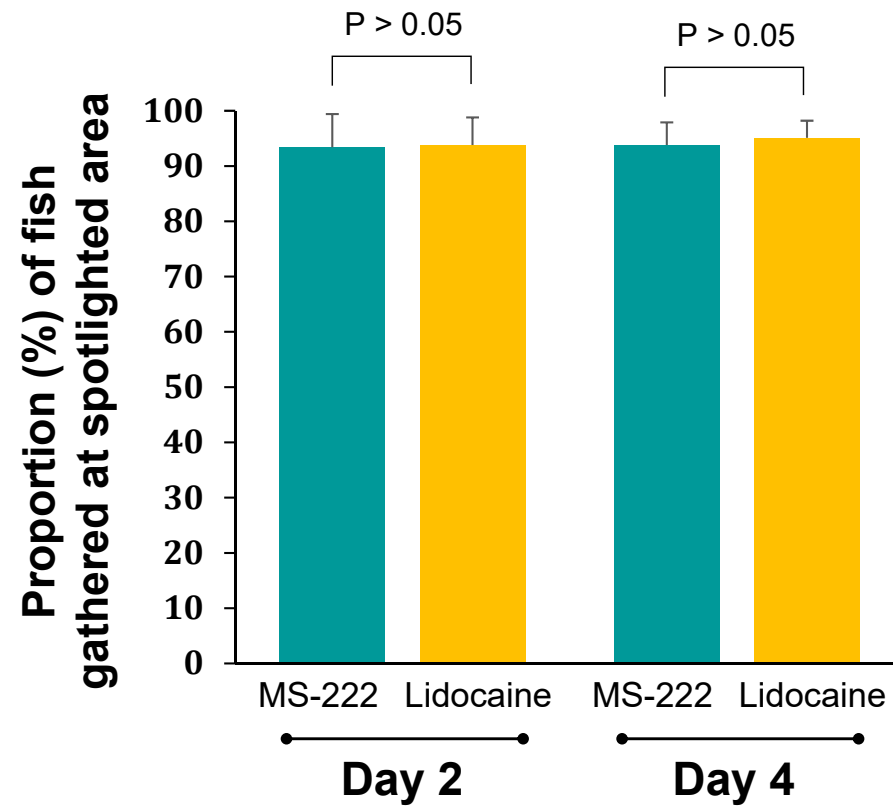

Supplement: S9 Fig — Positive phototactic behavior was examined in response to a spotlight under dark conditions on Day 2 and Day 4. There was no significant difference in phototactic characteristics of prolarvae anesthetized with MS-222 or lidocaine based on ANOVA and/or student’s t-test (P > 0.05). (PDF) [file pone.0209928.s009.pdf]
