# Supplementary material for: Anesthetic protocol for microinjection-related handling of Siberian sturgeon (Acipenser baerii; Acipenseriformes) prolarvae
Source: PLoS One. 2018 Dec 31;13(12):e0209928. doi: 10.1371/journal.pone.0209928 (PMC6312391; doi:10.1371/journal.pone.0209928)
Supplement: S10 Fig — Abbreviations are barbel rudiment (BR), brachial grooves (BG), external gills (EXG), myelencephalon cavity (MyC), olfactory pit (OP), and yolk sac (YS). Bars indicate 0.3 mm. (PDF) [file pone.0209928.s010.pdf]

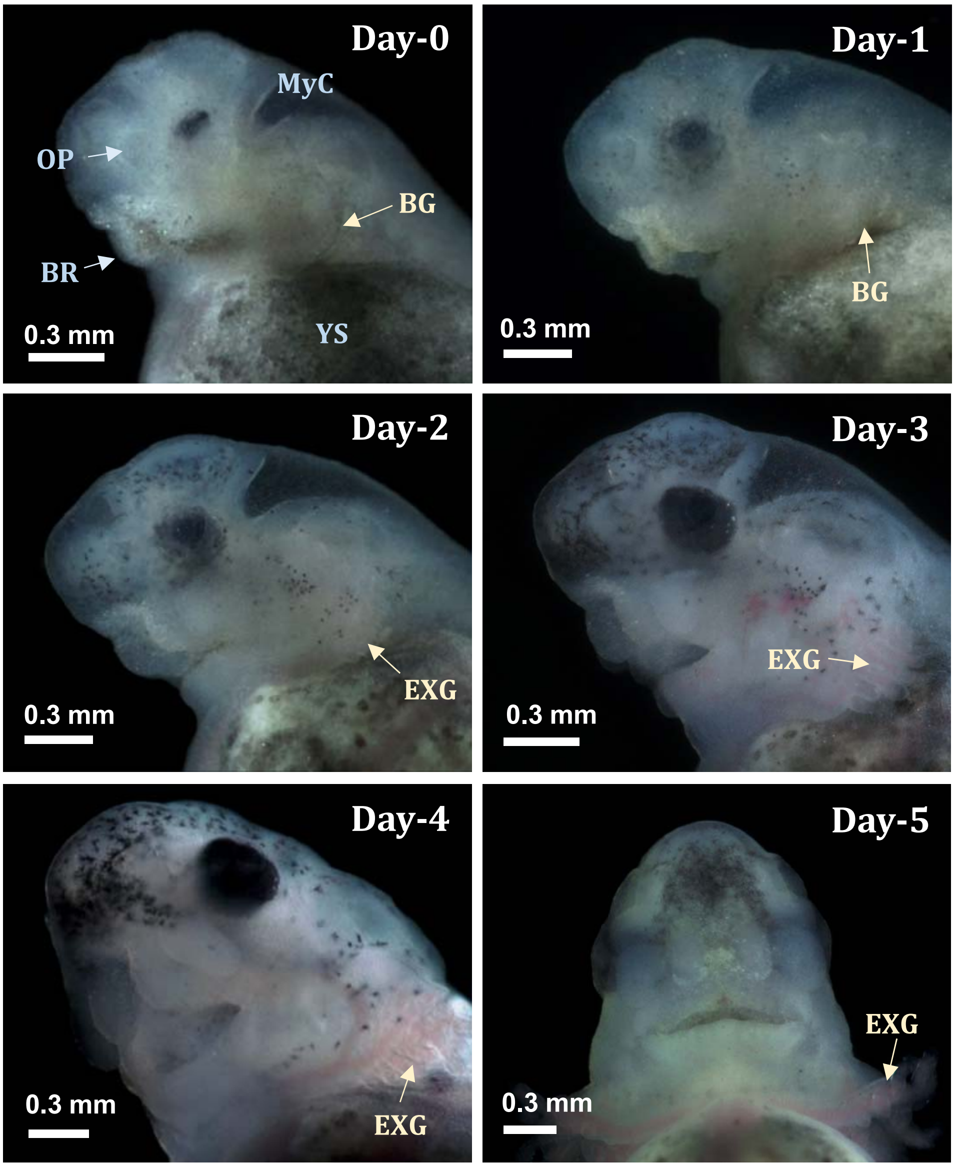

BR: barbel rudiment
BG: brachial grooves
EXG: external gills

MyC: myelencephalon cavity
OP: olfactory pit
YS: yolk sac
