# Supplementary material for: Anesthetic protocol for microinjection-related handling of Siberian sturgeon (Acipenser baerii; Acipenseriformes) prolarvae
Source: PLoS One. 2018 Dec 31;13(12):e0209928. doi: 10.1371/journal.pone.0209928 (PMC6312391; doi:10.1371/journal.pone.0209928)
Supplement: S1 Table — (PDF) [file pone.0209928.s011.pdf]

S1 Table. Quality parameters of water used for anesthetic/recovery experiments in this study

Parameter	Average level*
Dissolved oxygen	7.5 ± 0.5 mg/L
pH	7.4 ± 0.2 mg/L
Suspended solids	6.1 ± 1.0 mg/L
Total phosphorus	0.09 ± 0.02 mg/L
Nitrite	0.04 ± 0.01 mg/L
Nitrate	0.60 ± 0.02 mg/L
Ammonia nitrogen	< 0.01 mg/L
Hardness ($CaCO_3$)	32.1 ± 2.4 mg/L
Salinity	< 0.01%
Biochemical oxygen demand	1.2 ± 0.2 mg O_2/L

* Mean ± SD was based on at least triplicate measurements.
