# Supplementary material for: Anesthetic protocol for microinjection-related handling of Siberian sturgeon (Acipenser baerii; Acipenseriformes) prolarvae
Source: PLoS One. 2018 Dec 31;13(12):e0209928. doi: 10.1371/journal.pone.0209928 (PMC6312391; doi:10.1371/journal.pone.0209928)
Supplement: S2 Table — (PDF) [file pone.0209928.s012.pdf]

S2 Table. Average total length and body weight of Siberian sturgeon *A. baerii* prolarvae during the period Day 0 to Day 8 assessed in the present study

Age (Day)	Total length (mm)	Body weight (mg)
0	9.8 ± 0.3	13.8 ± 0.6
1	10.7 ± 0.4	14.4 ± 0.5
2	11.5 ± 0.4	16.1 ± 0.8
3	13.4 ± 0.4	16.6 ± 0.9
4	14.7 ± 0.5	19.2 ± 1.1
5	15.7 ± 0.3	22.5 ± 0.8
6	16.8 ± 0.5	25.2 ± 1.2
7	18.5 ± 0.5	30.1 ± 1.5
8	20.9 ± 0.6	35.5 ± 2.1

Mean ± SDs for length and weight at each age were measured with 48 randomly chosen prolarvae reared at 19 - 20 ºC.
