# Supplementary material for: Anesthetic protocol for microinjection-related handling of Siberian sturgeon (Acipenser baerii; Acipenseriformes) prolarvae
Source: PLoS One. 2018 Dec 31;13(12):e0209928. doi: 10.1371/journal.pone.0209928 (PMC6312391; doi:10.1371/journal.pone.0209928)
Supplement: S3 Table — (PDF) [file pone.0209928.s013.pdf]

S3 Table. Post-anesthesia/recovery viability of *A. baerii* prolarvae assessed in this study

Experiment	Prelarval age	Anesthetic agent	Anesthetic dose (mg/L)	No. prelarvae examined[1]	No. prolarvae survived				
					1 HPR[2]	6 HPR	12 HPR	24 HPR	Survival rate (%) at 24 HPR
**Experiment A**	Day-0	CON[3]	0	60	60	60	60	60	100.0
		Clove oil	50	60	60	60	60	60	100.0
			100	60	60	60	60	60	100.0
			200	60	60	60	59	59	98.3
			400	60	59	59	59	59	98.0
		Lidocaine	50	60	60	60	60	60	100.0
			100	60	60	60	60	60	100.0
			200	60	60	60	60	60	100.0
			400	60	60	59	59	59	98.3
		MS-222	50	60	60	60	60	60	100.0
			100	60	60	60	60	60	100.0
			200	60	60	60	60	60	100.0
			400	60	60	60	60	60	100.0
**Experiment B**	Day-0	CON	0	36	36	36	36	36	100.0
		MS-222	50	36	36	36	36	36	100.0
			100	36	36	36	36	36	100.0
			200	36	36	36	36	36	100.0
			400	36	36	36	36	36	100.0
**Experiment C**	Day-0	CON	0	50	50	50	50	50	100.0
		MS-222	100	50	50	50	50	50	100.0
			200	50	50	50	50	50	100.0

S3 Table. Continued

Experiment	Prelarval age	Anesthetic agent	Anesthetic dose (mg/L)	No. prolarvae examined[1]	No. prolarvae survived				
					1 HPR[2]	6 HPR	12 HPR	24 HPR	Survival rate (%) at 24 HPR
**Experiment D**	Day-0	CON[3]	0	60	60	60	60	60	100.0
		MS-222	200	60	60	60	60	60	100.0
	Day-1	CON	0	60	60	60	60	60	100.0
		MS-222	200	60	60	60	60	60	100.0
	Day-2	CON	0	60	60	60	60	60	100.0
		MS-222	200	60	60	60	60	60	100.0
	Day-3	CON	0	60	60	60	60	60	100.0
		MS-222	200	60	60	60	60	60	100.0
	Day-4	CON	0	60	60	60	60	60	100.0
		MS-222	200	60	60	60	60	60	100.0
	Day-5	CON	0	60	60	60	60	60	100.0
		MS-222	200	60	60	60	60	60	100.0
**Experiment E**	Day-1	CON	0	360	360	N/A[4]	N/A	359	99.7
		MS-222	200	360	360	N/A	N/A	360	100.0
	Day-2	CON	0	360	360	N/A	N/A	360	100.0
		MS-222	200	360	360	N/A	N/A	359	99.7
	Day-4	CON	0	360	360	N/A	N/A	358	99.5
		MS-222	200	360	360	N/A	N/A	359	99.7

[1], Total number of prelarvae from all the replicate groups
[2], HPR: hours post recovery
[3], CON: non-anesthetized control group (same handling without anesthetic)
[4], N/A: not assessed
